# Functional characterization of enhancer activity during a long terminal repeat's evolution

**DOI:** 10.1101/gr.276863.122

**Published:** 2022-10

**Authors:** Alan Y. Du, Xiaoyu Zhuo, Vasavi Sundaram, Nicholas O. Jensen, Hemangi G. Chaudhari, Nancy L. Saccone, Barak A. Cohen, Ting Wang

**Affiliations:** 1 Department of Genetics, Washington University School of Medicine, St. Louis, Missouri 63110, USA;; 2 The Edison Family Center for Genome Sciences and Systems Biology, Washington University School of Medicine, St. Louis, Missouri 63110, USA;; 3 Division of Biostatistics, Washington University School of Medicine, St. Louis, Missouri 63110, USA;; 4 Department of Developmental Biology, Washington University School of Medicine, St. Louis, Missouri 63110, USA

## Abstract

Many transposable elements (TEs) contain transcription factor binding sites and are implicated as potential regulatory elements. However, TEs are rarely functionally tested for regulatory activity, which in turn limits our understanding of how TE regulatory activity has evolved. We systematically tested the human LTR18A subfamily for regulatory activity using massively parallel reporter assay (MPRA) and found AP-1- and CEBP-related binding motifs as drivers of enhancer activity. Functional analysis of evolutionarily reconstructed ancestral sequences revealed that LTR18A elements have generally lost regulatory activity over time through sequence changes, with the largest effects occurring owing to mutations in the AP-1 and CEBP motifs. We observed that the two motifs are conserved at higher rates than expected based on neutral evolution. Finally, we identified LTR18A elements as potential enhancers in the human genome, primarily in epithelial cells. Together, our results provide a model for the origin, evolution, and co-option of TE-derived regulatory elements.

Changes in gene regulation have long been implicated as crucial drivers in evolution (King and Wilson 1975). Since the discovery of the SV40 enhancer element, enhancers have emerged as one of the major classes of *cis*-regulatory sequences that can modulate gene expression (Banerji et al. 1981; Moreau et al. 1981). Because of several unique properties, enhancers have emerged as excellent candidates upon which evolution can act. Enhancers are often active depending on cellular context like cell type or response to stimuli. This modularity can minimize functional trade-offs and allows selection to act more efficiently (Wray 2007). Furthermore, redundant enhancers, or “shadow” enhancers, provide robustness in gene regulatory networks and may allow for greater freedom to develop new functions (Hong et al. 2008; Cannavò et al. 2016).

The development of massively parallel reporter assays (MPRAs) has greatly accelerated our understanding of enhancers by facilitating simultaneous testing of thousands of DNA sequences (Patwardhan et al. 2009, 2012; Kwasnieski et al. 2012; Melnikov et al. 2012). MPRAs have been used to probe the enhancer potential of sequences underlying various epigenetic marks (Kwasnieski et al. 2014), dissect enhancer logic through tiling and mutagenesis (Melnikov et al. 2012; Ernst et al. 2016; Chaudhari and Cohen 2018), and decipher the effects of naturally occurring sequence variants (Patwardhan et al. 2012; Vockley et al. 2015; Tewhey et al. 2016; Ulirsch et al. 2016). Several studies have also used MPRA to understand the evolution of fly and primate enhancers, revealing widespread enhancer turnover (Arnold et al. 2014; Klein et al. 2018).

Transposable elements (TEs) are repetitive DNA elements that represent a rich source of genetic material for regulatory innovation (Feschotte 2008). In mammalian genomes, TEs have made substantial contributions to the collection of transcription factor binding sites (Wang et al. 2007; Bourque et al. 2008; Kunarso et al. 2010; Schmidt et al. 2012; Sundaram et al. 2014). These binding sites are often enriched within certain TE subfamilies, groups of similar TE sequences that are derived from a single ancestral origin. Individual copies of TE subfamilies can then be co-opted into gene regulatory networks such as in pregnancy and innate immunity (Lynch et al. 2011; Chuong et al. 2016). Overall, TEs make up a quarter of the regulatory epigenome in human (Pehrsson et al. 2019), and by some estimates, the majority of primate-specific regulatory sequences are derived from TEs (Jacques et al. 2013; Trizzino et al. 2017). Despite these advances in the field, there remains a gap in knowledge of how TEs obtain regulatory activity and how this activity changes over the course of evolution.

As repetitive sequences, TEs offer a unique perspective into the evolution of *cis*-regulatory elements. One intrinsic limitation for evolutionary studies is that each enhancer has one ortholog per species barring duplication or deletion, which constrains the sample size for analysis. Within a TE subfamily, each TE is descended from a common ancestor, with each copy evolving mostly independently. This provides a large sample size to draw upon within even a single genome. To serve as a representative subfamily, we selected LTR18A, which we previously identified to be enriched for MAF BZIP transcription factor K (MAFK) transcription factor binding peaks and motifs (Sundaram et al. 2014).

Here, we aim to investigate the evolution of regulatory potential in the LTR18A subfamily using MPRA. By using present-day LTR18A sequences found across seven primate species, we computationally reconstruct ancestral sequences during LTR18A evolution across a span of roughly 75 million years. We apply tiling and motif-focused approaches to test reconstructed and present-day LTR18A sequences for enhancer activity. Using natural sequence variations between LTR18A elements, we identify transcription factor binding sites that drive LTR18A enhancer activity, and validate them through mutagenesis. By annotating enhancer activity for the root and intermediate ancestral LTR18A elements in our reconstructed phylogenetic tree, we investigate the origin of enhancer activity for the LTR18A family as well as key mutations that have led to changes in activity over time. Finally, we explore the influence of selection on LTR18A and the possibility of co-option in the human epigenome.

## Results

### Reconstruction of the LTR18A phylogenetic tree

To reconstruct the evolutionary history of the LTR18A subfamily, we first identified high-confidence LTR18A elements in human and their orthologous elements in six other primate species. The LTR18A subfamily is found in the Simiiformes taxa (Storer et al. 2021). From the Simiiformes, we obtained RepeatMasker annotations for human (hg19), chimpanzee (panTro4), gorilla (gorGor3), gibbon (nomLeu3), baboon (papAnu2), rhesus macaque (rheMac3), and marmoset (calJac3) genomes. LTR18A elements between hg19 and GRCh38 differ by only 1 bp. Because of the similarity of the LTR18A, LTR18B, and LTR18C consensus sequences, we performed manual curation of hg19 LTR18A to select for LTR18A elements that are confidently assigned to the subfamily. Briefly, we filtered out LTR18A elements that could be aligned to either the LTR18B or LTR18C consensus, and we removed LTR18A elements that might be misannotated using paired LTRs (Supplemental Methods). Following these criteria, 181 out of 198 LTR18A elements annotated by RepeatMasker are retained (Supplemental Tables S1, S3). Next, we found primate orthologs for each hg19 LTR18A element by using synteny (Kuhn et al. 2013). Each hg19 LTR18A element with its primate orthologs was considered an ortholog set. We further selected for LTR18A pairs that have orthologs in chimpanzee, gorilla, and at least two of the four other primates. In the end, 46 (consisting of 23 pairs) LTR18A ortholog sets were chosen for ancestral reconstruction.

**Figure 1.**
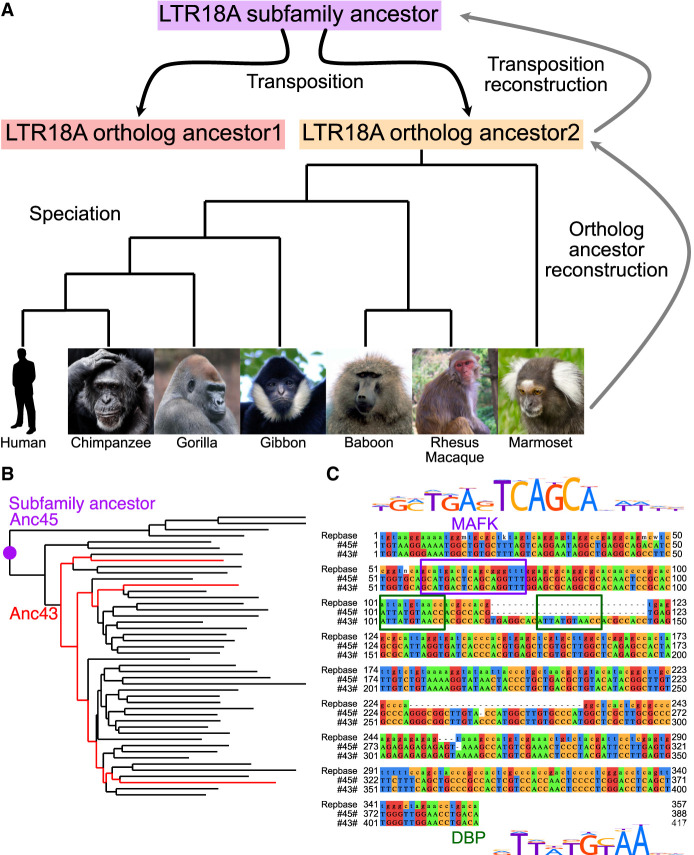
LTR18A ancestral reconstruction. (*A*) Model of LTR18A evolution split into transposition and speciation phases. Computational reconstruction was performed for ortholog ancestors and transposition intermediates using PRANK. (*B*) Phylogenetic tree for reconstructed transposition intermediates and ortholog ancestors at leaves. Ancestral node 43 (Anc43) is labeled in red, as well as the edges to ortholog ancestors that contain the 27-bp insert. The subfamily ancestor at ancestral node 45 (Anc45) is labeled by the purple dot. (*C*) Alignment of Repbase consensus (*top*), Anc45 (#45#, *middle*), and Anc43 (#43#, *bottom*). Motifs in the sequences are boxed. DBP is shown to represent CEBP-related motifs.

From our set of manually curated human LTR18A elements and their orthologs, we computationally reconstructed the LTR18A phylogenetic tree using a two-step process. Based on the unique characteristic of TEs to multiply by transposition and the presence of orthologous copies in different primate genomes, we split our reconstruction of LTR18A evolution into two phases corresponding to transposition and speciation (Fig. 1A). For each of the 46 sets of LTR18A orthologs, we aligned orthologs using MAFFT and then reconstructed ortholog ancestor and intermediate sequences using PRANK (Katoh et al. 2002; Löytynoja 2014). Then, using the ancestor sequences for the 46 LTR18A orthologs, we aligned and reconstructed the LTR18A subfamily ancestor as well as intermediates predating speciation (Methods). PRANK was chosen for ancestral sequence and phylogenetic tree reconstruction owing to its ability to model insertions and deletions. However, PRANK tends to be biased toward insertions in our reconstruction. Thus, we manually curated sequences following PRANK reconstruction for both ortholog ancestors and subfamily ancestors (Supplemental Methods).

Next, we evaluated our reconstructed LTR18A sequences to see if they are consistent with those derived from other methods. TE consensus sequences are often used as a representation of the ancestral state of the subfamily. Excluding insertions and deletions, our reconstructed LTR18A subfamily ancestor has an ∼5.9% substitution rate relative to the LTR18A consensus sequence, which is lower than the 16.1% subfamily average. This suggests that although we start from different elements and use different methodologies, both our reconstruction and the Repbase consensus are approaching each other. In addition to substitutions, our reconstructed ancestor also has ∼8.0% insertions compared with the consensus. The insertions appear to be caused by the consensus dropping bases if the majority of elements do not have the base in the alignment, as well as PRANK's tendency to include insertions when alignable sequence is present in more than one element. The MAFK motif enriched in LTR18A was present in both our reconstructed subfamily ancestor and the Repbase consensus. Overall, the topology of our reconstructed phylogenetic tree resembles the tree generated from all hg19 LTR18A elements (Supplemental Fig. S1). One feature of note occurs in node 43, two nodes from the root of the tree (Fig. 1B). Relative to the subfamily consensus sequence and our most ancestral reconstructed sequence at node 45, node 43 has a 27-bp insertion that contains a motif for one of the CCAAT-enhancer binding protein (CEBP)–related factors, D-box binding PAR bZIP transcription factor (DBP) (Fig. 1C). When we examined ortholog ancestor reconstructions for this insertion, three ortholog ancestors have an alignable 27-bp insert, and the insertion is present in all present-day primate orthologs (Supplemental Fig. S2). In hg19, 13/181 elements contain the insert. The insert-containing elements are spread throughout most of the hg19 LTR18A phylogenetic tree, which is consistent with a deep ancestral origin for the insert and occurrence in node 43 of our reconstruction. Additionally, we found that the CEBP motif is in the LTR18A consensus and enriched in the subfamily relative to genomic background (DBP log odds ratio 6.5). If the CEBP motif is functionally important, the insertion of a second CEBP motif could be an ancestral gain-of-function mutation. In conclusion, our reconstruction is able to generate a subfamily ancestor similar to the Repbase consensus and reveals evolutionary events that would otherwise be missed.

### Identification of important TFBS motifs in LTR18A enhancers

**Figure 2.**
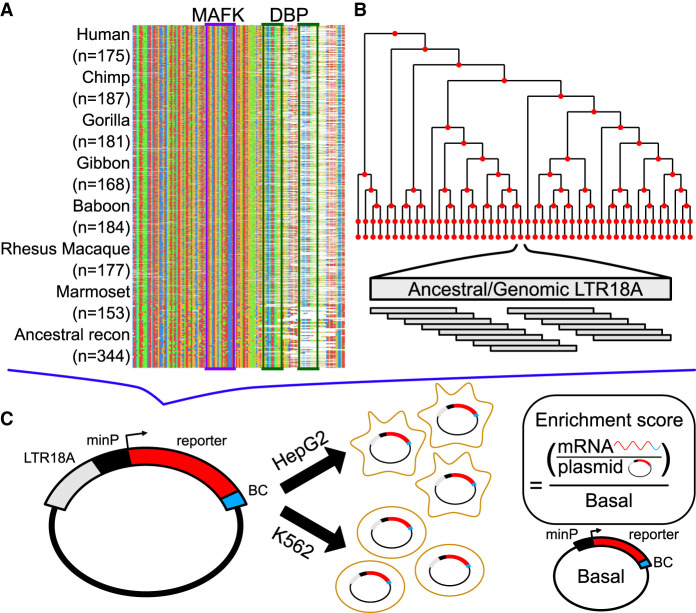
Schematic of MPRA. (*A*) Sequence alignment of motif-focused regions to test primate and ancestral reconstructed LTR18A elements. MAFK and DBP motif regions are boxed. (*B*) Tiling of ancestral and hg19 genomic LTR18A elements in a reconstructed phylogenetic tree. All elements were tiled with 160-bp tiles at 10-bp intervals. (*C*) Plasmid construct and enrichment score calculation. Each LTR18A fragment was integrated upstream of a minimal promoter (minP) and tagged with 10 unique barcodes (BCs) during library synthesis. The MPRA library was transfected into HepG2 and K562 cells. Enrichment scores are log_2_ ratios of RNA/DNA normalized to basal.

We designed our LTR18A MPRA library to assay elements at two resolutions for a total of 5664 tested LTR18A fragments (Methods) (Fig. 2). In one half, we synthesized motif-focused regions for 1225 LTR18A elements found across seven primate genomes, 280 ancestral reconstruction elements, and the Repbase consensus (Fig. 2A). Specifically, we took the sequence of each element aligning to the first 160 bp of our reconstructed ancestral node 43 (Methods). This allowed us to focus on the effects of sequence variation for both the MAFK motif and the CEBP motif. In the other half of the library, we synthesized 160-bp tiles at 10-bp intervals focused on testing all prespeciation ancestral reconstruction elements, ortholog ancestors, and present-day hg19 elements from our reconstructed phylogenetic tree (Fig. 2B). We cloned LTR18A motif-focused regions and tiles upstream of a pGL4 vector with the hsp68 promoter and then transfected the library of MPRA plasmids into cell lines to study the episomal enhancer effects of the LTR18A sequences, as is typical in classic reporter assays (Supplemental Methods; Fig. 2C).

**Figure 3.**
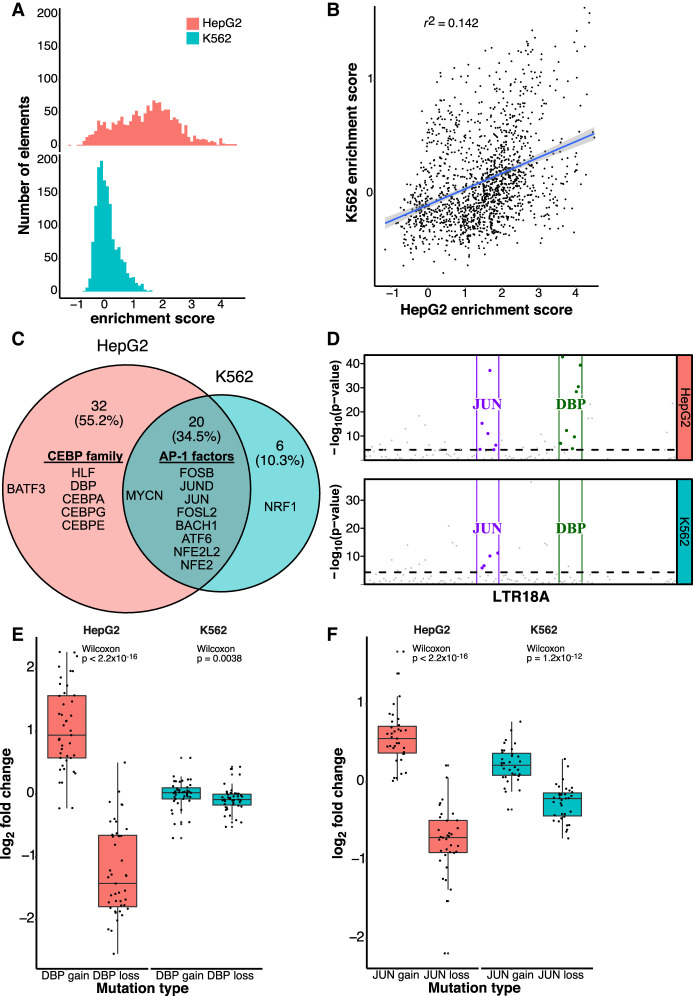
AP-1 motifs drive enhancer activity in HepG2 and K562, whereas CEBP motifs are HepG2 specific. (*A*) Distribution of enrichment scores of LTR18A motif-focused regions in HepG2 and K562. (*B*) Correlation of enrichment scores between HepG2 and K562. (*C*) Overlap of motifs significantly associated with active LTR18A. The top 10 transcription factor motifs in each cell line are displayed, with their placement in the Venn diagram determined by if the motif was found to be significant in one or both cell lines. AP-1- and CEBP-related transcription factors are grouped. (*D*) TEWAS significant nucleotides associated with active LTR18A. JUN and DBP motifs representing AP-1- and CEBP-related motifs are boxed. Significant positions (*P* < 5 × 10^−5^, *above* dotted line) within the two motifs that are associated with active elements are highlighted. (*E*) DBP mutagenesis effects on enhancer activity. (*F*) JUN mutagenesis effects on enhancer activity. *P*-values were derived from two-tailed Mann–Whitney *U* tests.

To understand cell type effects, we tested LTR18A for enhancer activity in HepG2 and K562 cell lines. We calculated enrichment scores for each element by taking the log_2_ of the RNA over DNA ratio followed by normalization to the basal hsp68 promoter. Normalizing to the basal promoter allowed us to have the same reference point between cell lines. Active elements were defined as those with enrichment scores greater than one, representing elements that increase transcription by more than twofold. When we compare the distribution of enrichment scores for HepG2 and K562, we find that LTR18A elements are generally more active in HepG2 than K562, which is consistent with cell type–specific activity commonly seen in enhancers (Fig. 3A). Out of 1506 motif-focused sequences tested, 1004 were classified as active in HepG2, whereas only 52 were classified as active in K562. For genomic LTR18A, 786 (123 from hg19) were active in HepG2 and 31 (four from hg19) were active in K562. Enrichment scores are positively but poorly correlated between HepG2 and K562 despite high correlations between biological replicates (*P* < 2.2 × 10^−16^) (Fig. 3B; Supplemental Fig. S3), implying differential sequence features required for enhancer activity between cell lines.

To identify important sequence features for enhancer activity, we took advantage of the natural sequence variation within LTR18A elements. Using AME motif enrichment analysis (McLeay and Bailey 2010), we asked if active elements were enriched for motifs compared with the rest of elements as background. Overall, 34.5% (20/58) motifs were enriched in active elements in both HepG2 and K562 (Fig. 3C). Of the shared motifs, activating protein 1 (AP-1)–related motifs from the JUN, FOS, and activating transcription factor/cyclic AMP-responsive element binding (ATF/CREB) families were in the top 10 most enriched for both cell lines. The top 10 most enriched motifs that were cell line–specific include the CEBP family motifs and BATF3 for HepG2 and NRF1 in K562. As an orthologous method, we investigated if individual nucleotide positions are associated with enhancer activity. As this is analogous to genome-wide association studies (GWAS) but focused on sequence variation within a TE subfamily, which we term TE-WAS, we adapted the GWAS tool PLINK to find significant nucleotides (Purcell et al. 2007; Chang et al. 2015). In HepG2, six of 11 JUN (AP-1 family) motif bases and eight of 11 DBP (CEBP family) motif bases are significantly associated with increased enhancer activity (Fig. 3D). In K562, after we adjusted our cutoff for active elements to be an enrichment score of at least 0.5 to increase the number of active elements from 52 to 239, four of 11 JUN motif bases and zero of 11 DBP motif bases are significantly associated with increased enhancer activity. In summary, both motif enrichment and TE-WAS approaches implicate AP-1 motifs as important to both HepG2 and K562 LTR18A enhancer activity, whereas CEBP-related motifs are HepG2 specific.

To validate the importance of CEBP and AP-1 motifs to enhancer activity, we created targeted mutations in the motif regions of LTR18A elements. We chose DBP to represent the CEBP family and JUN to represent the AP-1 family. We selected pairs of LTR18A orthologs in which one has the motif and the other does not by FIMO motif scanning (Grant et al. 2011). For elements with the motif, we mutated the motif bases to low information nucleotides based on the PWM. For elements without the motif, we changed the motif-aligned region to the consensus motif bases. To quantify the effect of motif mutations on enhancer activity, we took the log_2_ ratio of each motif-mutated LTR18A sequence to its native sequence (Fig. 3E,F). On average, DBP mutation gain and loss lead to a 2.07-fold increase and 2.36-fold decrease in enhancer activity, respectively, in HepG2. In contrast, the same DBP mutations have little effect in K562. JUN gain and loss lead to 1.49-fold increase and 1.68-fold decrease in HepG2 enhancer activity and 1.17-fold increase and 1.2-fold decrease in K562 enhancer activity. Both DBP and JUN mutagenesis results are consistent with our previous findings based on motif association.

### Evolution of LTR18A enhancer activity linked to sequence evolution

**Figure 4.**
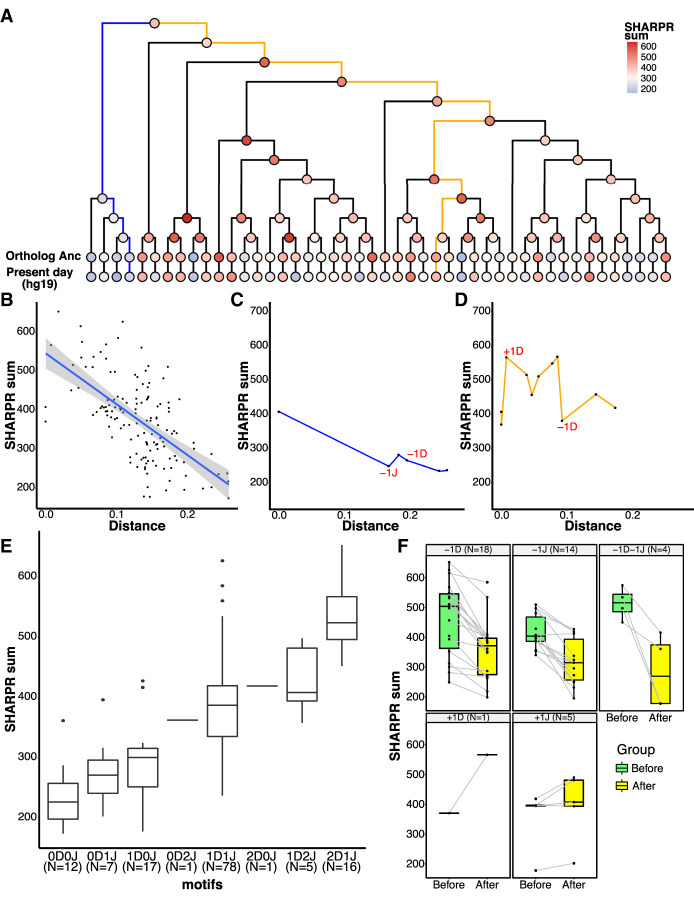
Evolution of regulatory activity in LTR18A in HepG2. (*A*) Phylogenetic tree of reconstructed ancestral LTR18A annotated at each node/element with the sum of SHARPR nucleotide activity scores. (*B*) Correlation of SHARPR sum and distance (substitution rate) from subfamily ancestor for each LTR18A in the phylogenetic tree. (*C*) Example of regulatory activity evolution along the blue path in *A*. Motif changes are labeled in red (D = DBP, J = JUN). (*D*) Same as *C*, but for the orange path in *A*. (*E*) Distribution of SHARPR sums for phylogenetic tree elements separated by DBP and JUN motif content. (*F*) Motif-associated changes in SHARPR sum. Each motif change in the phylogenetic tree is shown with the before and after motif change SHARPR sums connected by a line.

One of our primary goals was to understand how enhancer activity of LTR18A as a subfamily changed over time. To address this question, we synthesized 160-bp tiles at 10-bp intervals across each LTR18A ancestral sequence, ortholog ancestor, and hg19 element used in reconstruction (Fig. 2B). After obtaining enrichment scores, we estimated nucleotide activity scores across each element to infer their relative effects on enhancer activity using the SHARPR software for MPRA tiling designs (Ernst et al. 2016). Because of overall low activity in K562, we focus on HepG2 for evolutionary analysis. When examining nucleotide activity scores across the length of our reconstructed LTR18A subfamily ancestor, we observe regions of increased activity over basal. The CEBP and AP-1 motifs that we previously identified to be important for enhancer activity are embedded within the largest active region located near the start of the sequence (Supplemental Fig. S6). Across LTR18A elements of our reconstructed phylogenetic tree, we were able to confirm that regions of increased SHARPR nucleotide activity were enriched for CEBP and AP-1 motifs (Supplemental Table S5). As SHARPR nucleotide activity scores could discover the same biologically meaningful sequences as our previous analyses, we took the sum of activity scores across each LTR18A element and annotated them in our tree (Fig. 4A). From a broad perspective, we were able to make several observations. First, the most divergent (leftmost) lineage on the tree loses enhancer activity early, and enhancer activity throughout the lineage remains low to the present day (Fig. 4C). This low activity lineage contrasts with the rest of the tree, where evolutionary intermediates show relatively high activity followed by less active elements at the ortholog ancestor and present-day elements. Indeed, the overall trend appears to be that enhancer activity decreases over time, as shown by the decrease in SHARPR sum with increasing divergence from the LTR18A subfamily ancestor (Fig. 4B). On the other hand, there is an increase in activity in the middle lineages, some of which persists to the ortholog ancestors and present-day elements (Fig. 4D). Finally, the enhancer activity of present-day hg19 LTR18A elements and their corresponding ortholog ancestors is positively correlated with mostly small differences in activity, implying that postspeciation evolution has had small effects on regulatory potential overall (Supplemental Fig. S7).

To further investigate why enhancer activity changes in our LTR18A tree, we looked at differences in CEBP and AP-1 motif presence using DBP and JUN as representatives. When elements are categorized by the number of DBP and JUN motifs, the number of motifs is positively correlated with SHARPR sum (Fig. 4E). Furthermore, DBP or JUN loss correlates with a decrease in SHARPR sum, with rare motif gains generally corresponding to increased SHARPR sums (Fig. 4F). Because of the significance of the DBP motif, we evaluated ancestral node 43 as the sole evolutionary intermediate that gained a second motif through an insertion event (Fig. 1B). The motif gain leads to an increase in SHARPR sum of ∼39%, which is similar to the average effect size of the DBP motif (∼38%). This effect is validated by mutagenesis of our LTR18A subfamily ancestor and consensus to have the same 27-bp insertion (34% and 32% increase, respectively), as well as ablation of the second DBP motif in ancestral node 43 (41% decrease). In summary, sequence evolution, especially at the CEBP and AP-1 motifs, directly affects the ability of LTR18A to act as regulatory elements, and most mutations have led to a decrease in regulatory potential.

### Evidence of purifying selection for enhancer-associated CEBP and AP-1 motifs

Given that LTR18A has regulatory potential in certain cellular contexts like HepG2, we explored the possibility of host exaptation through the lens of selection. We first asked if LTR18A elements in chimpanzee, gorilla, gibbon, baboon, rhesus macaque, and marmoset have increased substitution rates compared with their human orthologs with respect to the distance between genomes. On average, LTR18A orthologs have slightly elevated substitution rates (12%–32%) than the corresponding genomes (Supplemental Table S2). The increased substitution rate holds true even when only considering masked regions of the genome. Although it is possible that the genomic background rate includes regions under selection, the LTR18A substitution rates across primate species are overall inconsistent with purifying selection for the subfamily. Furthermore, both phyloP and phastCons scores at LTR18A elements provide no evidence of selection at the subfamily level across 30 mammals, including 27 primates (Supplemental Fig. S8; Siepel et al. 2005; Pollard et al. 2010).

**Figure 5.**
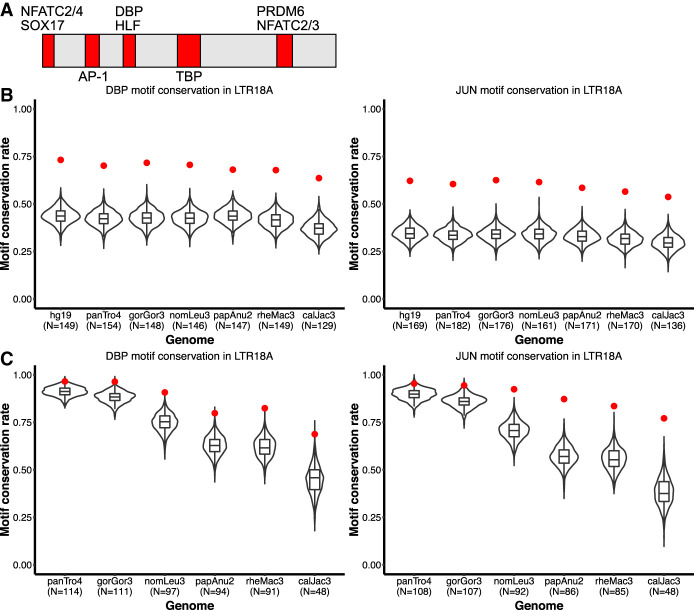
DBP and JUN motifs are more conserved than expected. (*A*) Motifs that are fully encompassed within shared, conserved 10-bp sliding windows across seven primate species. Motif locations in red are relative to the LTR18A Repbase consensus sequence. (*B*) Distribution of expected neutral DBP and JUN motif conservation rates from the consensus motif across primate species. One thousand simulations are displayed for each species. The observed conservation rate is shown by the red point. (*C*) Same as *B*, but for conservation rates from the hg19 ortholog as reference.

Although there is no evidence that LTR18A as a whole is under purifying selection, it is possible that certain regions within LTR18A are. We aligned LTR18A elements in each of our seven primate species to the LTR18A consensus and tested sliding 10-bp windows for increased conservation compared with the average window. Overall, 29% (707/2429) of all 10-bp windows are significantly more conserved than the average window. The majority (84%) of conserved 10-bp sliding windows are shared across all seven primates for a total of 24.5% (85/347) possible 10-bp windows covering 58% of the LTR18A consensus (208/357 bp) being classified as conserved. Shared, conserved regions defined by our sliding window analysis contain transcription factor motifs, including AP-1 and CEBP (Fig. 5A).

Because CEBP and AP-1 motifs are critical for enhancer activity, we hypothesized that the motifs provided by LTR18A have been under purifying selection and, consequently, show higher conservation than expected under a neutral model of evolution. To obtain the background motif conservation rates, we adapted a method previously used in yeast (Doniger et al. 2005). Briefly, we take the sum of probabilities for all sequences that match a motif PWM, with each sequence probability calculated starting from the LTR18A consensus and the observed transition and transversion rate of the LTR18A subfamily. As in previous analyses, we chose DBP and JUN to represent CEBP and AP-1. Expected conservation rates for DBP and JUN are consistent across species, ranging from 38.7% in marmoset to 44.8% in human for DBP and 34.1% in marmoset to 39.3% in human for JUN (Table 1). Meanwhile, observed DBP and JUN conservation rates are on average 69.3% and 59.3%, respectively, which is 26.4% and 21.6% higher than expected. This indicates that CEBP and AP-1 motifs from the ancestral LTR18A sequence are being retained and may be under selection. Measuring conservation from the LTR18A consensus includes the transposition phase of TE evolution, which could select for CEBP and AP-1 motifs owing to enhancing transcription of the ERV. To address conservation specifically during primate evolution, we recalculated conservation rates by comparing human LTR18A elements to their primate orthologs. Generally, DBP and JUN motifs are significantly more conserved than expected (Table 2). The one exception is JUN for the human–chimpanzee comparison, which might be owing to low human–chimpanzee divergence. We also confirmed higher motif conservation rates during transposition + speciation and speciation phases using simulations based on observed transition and transversion rates (Fig. 5B,C). Together, our analysis suggests that CEBP and AP-1 motifs contributed by LTR18A have been under purifying selection in primates both before and after speciation.

**Table 1.**
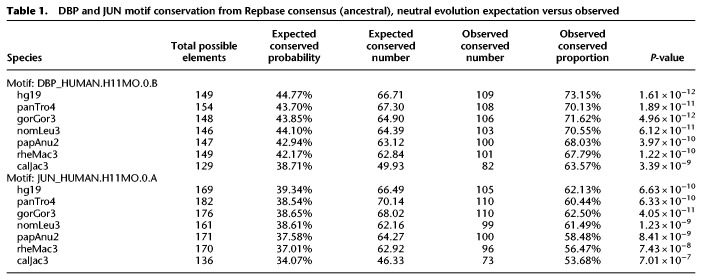
DBP and JUN motif conservation from Repbase consensus (ancestral), neutral evolution expectation versus observed

**Table 2.**
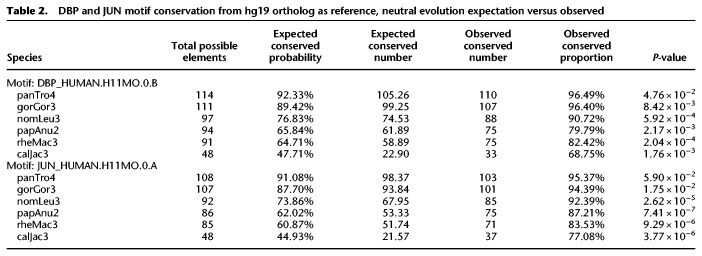
DBP and JUN motif conservation from hg19 ortholog as reference, neutral evolution expectation versus observed

### Human LTR18A has epigenetic signatures of active regulatory elements

Our MPRA reveals that LTR18A elements have the sequence features to be activating regulatory elements depending on cellular context. To explore the relationship between regulatory potential from MPRA and enhancer function in the genome, we examined epigenetic marks in HepG2 and K562 using ENCODE data (The ENCODE Project Consortium et al. 2020). We first profiled LTR18A elements overlapping ATAC peaks for open chromatin, which is a common epigenetic feature for active regulatory elements. In HepG2, LTR18A is not enriched for ATAC peaks, with only five LTR18A elements overlapping with peaks. On the other hand, K562 has 11 overlapping LTR18A elements. This contrasts with the high MPRA activity in HepG2 relative to K562. Additionally, H3K27ac and H3K4me1, histone marks commonly associated with active enhancers, are also low across LTR18A in HepG2 and K562 (Supplemental Fig. S9). Altogether, the overall lack of active epigenetic marks at LTR18A in HepG2 and K562 implies that they are largely inactive as regulatory elements in the two cell lines, despite many showing enhancer activities in reporter gene assays. We hypothesized that epigenetic repression of LTR18A may be the cause for the lack of active enhancer marks in HepG2. Consistent with this hypothesis, repressive histone mark H3K9me3 is enriched over LTR18A compared with the surrounding genomic region, with the peak in signal possibly indicating that LTR18A is targeted for silencing (Supplemental Fig. S9). These results suggest that although LTR18A elements possess the sequence features necessary for enhancer activity, they can be epigenetically silenced.

**Figure 6.**
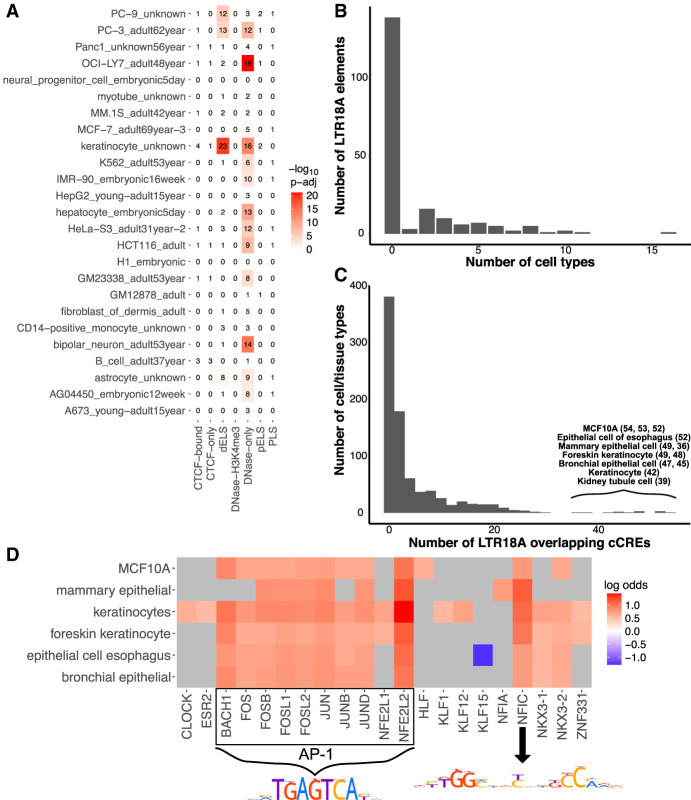
LTR18A elements are associated with enhancer epigenetic marks in human. (*A*) Overlap of LTR18A with ENCODE cCREs across 25 full-classification cell/tissue types. (dELS) Distal enhancer-like signature; (pELS) proximal enhancer-like signature; (PLS) promoter-like signature. The number of elements that overlap with cCREs are shown as well as their −log_10_-adjusted *P*-value by BEDTools Fisher. (*B*) Distribution of LTR18A elements overlapping cCREs across multiple full-classification cell/tissue types. (*C*) Distribution of cell/tissue types overlapping LTR18A elements. The top cell/tissue types are displayed with the number of LTR18A elements that overlap with a cCRE. (*D*) Motifs associated with the cCRE-overlapping LTR18A elements from the top cell/tissue types in *C*. Gray indicates nonsignificance at an adjusted *P*-value threshold of 0.05. PWMs for JUN (AP-1-related factors) and NFIC are shown.

Although most of the LTR18A subfamily is unlikely to be active in HepG2 and K562, we sought to ascertain the contribution of LTR18A to the regulatory genome across human cell types and tissues. To get a global perspective, we overlapped LTR18A elements with candidate *cis*-regulatory elements (cCREs) as defined by ENCODE Registry V2 across 839 cell/tissue types (The ENCODE Project Consortium et al. 2020). Despite the limited number of cell/tissue types (25) that have full classification of cCREs, 69 of 198 (34.8%) LTR18A elements overlap with a cCRE, most of which (87%) have enhancer-like signatures (ELSs) in at least one cell/tissue type. This represents 29.3% of all LTR18A bases, which is about 3.1× enriched over the genomic background (*P* < 3.5 × 10^−10^, BEDTools Fisher). Among fully classified cell/tissue types, keratinocytes have the highest number of LTR18A elements associated with ELS, followed by the PC-3 and PC-9 cell lines (Fig. 6A). LTR18A is not restricted to a single cell/tissue type, as some LTR18A elements are associated with cCREs in multiple cell/tissue types (Fig. 6B). Across all 839 cell/tissue types, cell types with the most LTR18As overlapping cCREs largely consist of epithelial cells, such as MCF10A, mammary epithelial cells, esophagus epithelial cells, and foreskin keratinocytes (Fig. 6C). To corroborate cCRE results that are based on DNase I hypersensitivity, H3K27ac, H3K4me3, and CTCF ChIP-seq, LTR18A elements were intersected with ENCODE ATAC-seq peaks across 46 cell/tissue types. Similar to cCREs, LTR18A is especially enriched for ATAC peaks in the epithelial cells/tissues foreskin keratinocytes and esophagus mucosa (11.4× and 16.1× enrichment over background, respectively; BEDTools Fisher). Although certainly not comprehensive, the available epigenetic data support an active enhancer-like state for LTR18A with the highest enrichment in epithelial cells.

As LTR18A enhancer potential is influenced by sequence variation, especially at transcription factor binding sites, we sought to understand whether transcription factor motifs are associated with active epigenetic states. Without considering cell/tissue type, we found only the AP-1-related FOSL1 and FOSL2 transcription factor motifs to be significantly associated with LTR18As overlapping cCREs relative to other LTR18As. Because of the cell type–specific nature of most enhancers, we further examined motifs enriched in cCRE-associated LTR18A in the top cell/tissue types (Fig. 6D). Many of the most common motifs are of AP-1 transcription factors. Another common motif is NFIC, which is consistent with an activating role previously described in cancer and could serve a similar role in activating LTR18A elements (Fane et al. 2017). Of note, the CEBP-related factor HLF is enriched only in the MCF10A cell line. Using ATAC data, we confirmed AP-1 and NFIC motifs as enriched in LTR18A elements associated with active epigenetic states in foreskin keratinocytes and esophagus mucosa. Altogether, these results suggest that LTR18A elements become epigenetically activated in epithelial cells primarily through AP-1 transcription factors and NFIC.

## Discussion

Since Britten and Davidson (1971) first hypothesized how repetitive elements could influence the development of gene regulatory networks, a growing number of studies have shown the contribution of TEs as regulatory modules. Using LTR18A as a representative subfamily, we performed the first systematic functional testing of regulatory potential for a TE subfamily using a MPRA. By taking advantage of the natural sequence variation across elements, we identify AP-1- and CEBP-related motifs as important drivers of LTR18A regulatory activity. This regulatory activity is highly dependent on cell context, with LTR18A displaying much higher activity in HepG2 than in K562. However, the sequence potential for regulatory activity does not necessarily reflect activity in the genome, as shown by LTR18A elements rarely associating with active epigenetic marks in HepG2. Because of the general repression of TEs, we believe that similarly silenced TEs with the potential for enhancer activity may be common. These inactive TEs may be latent under epigenetic control, but there remains the possibility that a changing epigenome, such as during tumorigenesis, can reactivate them (Jang et al. 2019).

Another unique aspect of this study is leveraging the phylogenetic relationship between LTR18A elements within human and across primate species to investigate the origin and evolution of regulatory activity in the subfamily. Previous research has implicated two evolutionary paths through which TE sequence can contribute to the spread of regulatory modules. The first case is when the ancestral TE originally possesses the driving regulatory features, such as the TP53 binding site in LTR10 and MER61 or the STAT1 binding site in MER41B (Wang et al. 2007; Chuong et al. 2016). A second possibility exists in which the ancestral TE gains the regulatory module in one lineage through mutation before amplification, such as the 10-bp deletion in ISX relative to ISY in *Drosophila miranda* that recruits the Male Specific Lethal complex (Ellison and Bachtrog 2013). In the LTR18A family, we observe both scenarios. Both CEBP and AP-1 motifs are found in the LTR18A consensus and our reconstructed subfamily ancestor, and many elements retain the motifs to the present day. Divergence from the ancestor over time, especially at the two motifs, is correlated with a decrease in regulatory activity. In addition to the two consensus motifs, a second CEBP motif is gained through an insertion at an early evolutionary timepoint. This second CEBP motif further increases the regulatory potential of LTR18A. Ultimately, however, few present-day elements have maintained the second motif. This could be explained by negative selection or a deletion bias from the sequence similarity of the insertion with the upstream sequence. It is also plausible that our evolutionary reconstruction makes an incorrect assumption about the timing of the second CEBP motif, and each one occurred independently rather than through a common ancestor. Under this scenario, a potential mechanism for the recurrent insertions is that the region is prone to replication slippage during replication or transposition, resulting in multiple independent duplications of the CEBP motif.

An intriguing possibility is the relationship between TE regulatory potential and genomic expansion. In our reconstructed LTR18A phylogenetic tree, we observe loss of enhancer activity in the leftmost lineage going as far back as its lineage ancestor. This low enhancer activity lineage corresponds to the earliest diverging branch in the human LTR18A subfamily phylogenetic tree and composes only about one of six (27/181) of all elements. On the other hand, the major lineage of LTR18A has enhancer activity throughout transposition. The stark contrast between the two lineages in enhancer activity and abundance leads us to speculate that the regulatory potential of LTR18A was directly related to its ability to expand in the genome, a hypothesis with which our data are consistent (Supplemental Fig. S10). This is perhaps unsurprising, as transcription is typically the first step of transposition and provides the substrate for integration of retrotransposons. However, one important consequence is that transcription factor binding sites that contribute to TE regulatory potential could be enriched within a subfamily owing to biased lineage amplification. This appears to have been the case for the recently reclassified LTR7 subfamilies, each of which possess a unique set of transcription factor motifs and underwent a wave of genomic expansion to fill different early embryonic niches (Carter et al. 2022). It will be important for future studies to distinguish between selection and passive enrichment of transcription factor binding sites through lineage amplification.

To compare ancestral and present-day LTR18A elements, we tested all elements within the same cell line using a MPRA. This assumes that HepG2 and K562 cells provide the same *trans* environment as the equivalent primate and ancestral cell types. Previous studies suggest that transcription factor binding and subsequent activation of transcription are deeply conserved from humans to flies (Nitta et al. 2015; Stampfel et al. 2015). Klein et al. (2018) make a similar assumption in their study of liver enhancer evolution in primates and find the same general trend that present-day elements have lost enhancer activity relative to the ancestral state. Another potential caveat is the episomal nature of the MPRA design, which takes LTR18A out of its native chromatin context. MPRA studies comparing the regulatory effects at different genomic loci and comparing episomal and lentiviral integration contexts have generally shown that the relative enhancer activities seen on episomal plasmids are similarly reflected compared with those integrated into the genome (Maricque et al. 2019; Klein et al. 2020). However, this remains to be confirmed for TEs, which could be subject to regulatory restraints targeting repetitive elements.

Most TEs are thought to be under neutral evolution and do not significantly impact phenotype. We find that LTR18A elements as a whole have higher mutation rates than the genomic average and do not show signs of selection based on phyloP and phastCons scores. Despite the lack of evidence for selection at the element level, AP-1 and CEBP binding motifs found within LTR18A are more conserved than expected under the neutral model of evolution. This suggests that selection does not need to apply to entire TEs and instead acts on functional units found within each element. Indeed, we find that at least a third of LTR18A elements have enhancer-associated epigenetic marks, and in some cell/tissue types, the active elements are enriched for the conserved AP-1 motif. Although the CEBP motif is not significantly enriched with active elements outside of MCF10A, we suspect that the motif is important in other cell/tissue types that have yet to be profiled.

## Methods

### LTR18A ancestral reconstruction

To find LTR18A ortholog sets for ancestral reconstruction, we searched for LTR18A element pairs that fulfilled several requirements. First, the hg19 LTR18A elements must have orthologs in chimpanzee and gorilla. Second, elements must have orthologs in at least two of the other primate species: gibbon, baboon, rhesus macaque, and marmoset. Third, hg19 LTR18A elements must be >250 bp (>70% of consensus) in length. Finally, both elements of a pair need to pass all requirements to be selected for ancestral reconstruction. Orthologs were defined using the chain files from UCSC to find LTR18A elements within the same syntenic blocks (Kuhn et al. 2013). LTR18A elements that correspond with multiple orthologs in the same genome, or vice versa, were excluded.

Ancestral reconstruction of both ortholog ancestors and subfamily ancestors used MAFFT and PRANK followed by manual curation (Katoh et al. 2002; Löytynoja 2014). To generate ortholog ancestors, we aligned ortholog sets (e.g., human, chimpanzee, gorilla, gibbon, baboon orthologs) using MAFFT multiple sequence alignment. We used the alignments to produce ancestral and intermediate sequences as well as the phylogenetic tree using PRANK. The PRANK phylogenetic trees typically reflected the expected evolutionary relationship between the seven primate species. Next, we manually adjusted ortholog ancestors to remove unlikely insertions (Supplemental Methods). After manual curation of ortholog ancestors, we used MAFFT and PRANK to reconstruct the phylogenetic tree and sequences of LTR18A subfamily ancestral sequences.

### LTR18A MPRA library design

The MPRA library was designed to consist of a motif-focused half and a tiling half. To design the motif-focused half of our MPRA library, we took advantage of the relatedness of TEs within the same subfamily. Similar to RepeatMasker, we can align all LTR18A elements to a reference sequence. Instead of using the subfamily consensus sequence, we used our reconstructed ancestral node 43 to perform pairwise global alignments to all present-day and reconstructed elements. Then, we took the sequence of each element aligned to the first 160 bp of ancestral node 43. We filtered out elements that have <70 bp owing to deletions and elements that have >160 bp owing to insertions. We also removed elements that contain a restriction site that we used for cloning. In total, 1225/1387 RepeatMasker-annotated LTR18A elements across seven primate genomes, all 280 reconstructed elements, and the Repbase consensus sequence were included. For the tiling half of the library, we selected all prespeciation ancestral reconstruction elements, ortholog ancestors and their present-day hg19 elements, 11 additional hg19 elements, and the LTR18A consensus. We then synthesized 160-bp tiles at 10-bp intervals spanning each selected element for a total of 3236 fragments. In addition to motif-focused and tiled sequences, we selected 456 elements for reverse complements (Supplemental Fig. S4), 37 pairs of elements for JUN mutagenesis, and 46 pairs of elements for DBP mutagenesis. Elements for mutagenesis were chosen based on the closest primate ortholog with/without the motif. JUN motifs were mutated to TCACCAATGGT, and DBP motifs were mutated to TCCCACAGCAT. Non-motif-containing elements were mutated to GCTGAGTCATG for JUN and ATTATGTAACC for DBP. We also made DBP and JUN mutations in ancestral node 45 and 43 and the Repbase consensus, resulting in seven additional mutated motif-focused and 168 tiled sequences. For positive and negative controls, we selected 223 regions from a previous study by Ernst et al. (Supplemental Fig. S5; Ernst et al. 2016). Thirty dinucleotide shuffled LTR18A Repbase consensus sequences were included as a second set of negative controls (Bailey et al. 2015). Each sequence was tagged with 10 unique barcodes during synthesis. To control for differences in overall library activity between cell lines, we included a set of sequences that would leave only the basal hsp68 promoter tagged with 300 barcodes. In total, 5918 elements were synthesized using 59,470 unique barcodes.

### LTR18A MPRA enrichment score calculation

For each tested element, we added up read counts for all of its barcodes and filtered out those with fewer than five total counts in any of three transfection replicates or DNA input. Reads were then normalized to counts per million (CPM). Expression of an element was calculated as RNA CPM/DNA CPM. Expression was normalized to the average of basal construct transfection replicates. Finally, enrichment score was calculated as the log_2_ of normalized expression. Enrichment scores of elements are provided in Supplemental Data S1.

### Transcription factor motif enrichment

LTR18A sequences were separated into active and inactive groups depending on the enrichment score in HepG2 and K562. AME motif enrichment was performed to find motifs enriched in active LTR18A over inactive LTR18A using an *E*-value threshold of 0.05 (McLeay and Bailey 2010; Kulakovskiy et al. 2018). All motifs that were enriched are listed in Supplemental Table S4.

### TE-WAS analysis of nucleotides and motifs

LTR18A sequences were globally aligned pairwise to the ancestral node 43 sequence as reference. Pairwise alignments were then combined based on the common reference. Positions that had bases (not gaps) in <20% of all LTR18A sequences were removed. This filter retained all consensus base positions.

GWAS analysis tool PLINK was used to identify nucleotides significantly associated with the phenotype, such as MPRA activity/inactivity or ATAC peak (Chang et al. 2015). We limited tested nucleotides at each position to the most common nucleotide at the position across LTR18A sequences to give us greater confidence based on sample size. We ran PLINK association analysis using the above-described alignment and MPRA active/inactive annotations for each element based on enrichment score. Nucleotides were deemed significant if *P*-value < 5 × 10^−5^.

From the list of significant nucleotides in TE-WAS, we identified transcription factor motifs from the core human HOCOMOCOv11 database that are overrepresented based on information content (Kulakovskiy et al. 2018). Information content at each significant nucleotide was calculated from each motif's position frequency matrix with the background nucleotide frequencies of 0.25. The information content of significant nucleotides within each motif was then compared with a background expectation derived from 1000 random shuffles of significant nucleotides for the phenotype. Motifs were identified if they had higher information content from significant nucleotides than background using *t*-test and more than significant nucleotides within the motif.

### Evolutionary analysis using SHARPR

From tiled MPRA, we calculated regulatory activity for full-length elements using SHARPR with a few adjustments (Ernst et al. 2016). For each tile of an element, the previously calculated enrichment score was used as input for SHARPR inference with the default varpriors of 1 and 50. Each inferred 10-bp step was then normalized to the mean inferred value for randomly shuffled basal elements as background. The SHARPR combine and interpolate commands were used to generate the SHARPR nucleotide activity scores. Finally, full-length element activities were calculated as the sum of nucleotide scores across each element.

To validate the SHARPR approach, we identified motifs that were enriched in peaks, or regions of high nucleotide activity. Peaks were defined as regions with nucleotide activity scores greater than three standard deviations above the basal mean. Enriched motifs were then identified in peak regions using AME using shuffled sequence as background (McLeay and Bailey 2010).

### Transcription factor motif conservation

For sliding window conservation analysis, we aligned all present-day genomic LTR18A elements to the Repbase consensus sequence using the previously defined method. Conservation, defined as the percentage match to the consensus, was calculated for each 10-bp window for each element in each species. Windows with gaps or degenerate bases in at least half of the total window length (five or more) were excluded. The mean conservation was then calculated for each 10-bp window separately for each species. Windows were determined to be significantly conserved using *t*-test comparing conservation across elements in the window against conservation across all windows, with a *P*-value threshold of 0.05 after Bonferroni correction. Only windows that were conserved in all seven primate species were kept for further analysis. Motif scanning by FIMO was performed to find transcription factor motifs fully within conserved windows (Grant et al. 2011).

For JUN and DBP transcription factor motif conservation analysis, transition and transversion rates in the LTR18A subfamily were calculated for each species. The neutral expectation for motif conservation was calculated as previously described (Doniger et al. 2005). We identified all *k*-mers of the motif length that are found by FIMO (Grant et al. 2011). The total motif conservation probability was calculated as the sum of the probabilities for each motif *k*-mer. We used the Repbase consensus sequence as the ancestral LTR18A state. To represent postspeciation conservation, we used hg19 orthologs as the reference to compare with other primate LTR18A elements. The observed motif conservation rate was calculated for each species based on the percentage of elements that retain the motif. Elements with gaps in the alignment to its reference were excluded. Statistical significance was determined by one sample test of proportions and a *P*-value threshold of 0.05. We also simulated transcription factor motif conservation rates for each primate species. Each simulation consisted of randomly mutating nucleotides in the motif region of each LTR18A element based on the observed transition and transversion rates. One thousand simulations were performed for each motif.

### Overlap of LTR18A with genomic annotations

The cCRE genome annotations and various epigenetic data sets such as ATAC-seq, histone ChIP-seq, and WGBS were downloaded from ENCODE (https://www.encodeproject.org) (The ENCODE Project Consortium et al. 2020). The phyloP and phastCons scores were downloaded from UCSC and converted to bedGraph (Kuhn et al. 2013). Overlaps with LTR18A elements were obtained by BEDTools intersect with the criteria of at least 50% LTR18A length overlapping with a cCRE or epigenetic mark peak (Quinlan and Hall 2010). Enrichment of LTR18A in cCREs and ATAC peaks was obtained by BEDTools Fisher using the same criteria. Heatmaps at and around LTR18A were generated using deepTools (Ramírez et al. 2016). LTR18A elements with HepG2 MPRA and ATAC peak overlap annotations are shown in Supplemental Table S6. Accession codes for publicly available data sets used in this study are listed in Supplemental Data S2.

### Identification of motifs associated with cCRE-overlapping LTR18A

Fisher's exact test was used to determine if transcription factor binding motifs in LTR18A elements are associated with cCRE overlap. Motifs that had *P*-values below 0.05 after correcting for number of motifs tested were considered significant. The top six cell/tissue types were selected for analysis as they provided the greatest number of LTR18A elements overlapping cCREs.

## Data access

All raw and processed sequencing data generated in this study have been submitted to the NCBI Gene Expression Omnibus (GEO; https://www.ncbi.nlm.nih.gov/geo/) under accession number GSE201068. All custom scripts are available in Supplemental Code.

## Supplementary Material

Supplemental Material

## References

[GR276863DUC1] Arnold CD, Gerlach D, Spies D, Matts JA, Sytnikova YA, Pagani M, Lau NC, Stark A. 2014. Quantitative genome-wide enhancer activity maps for five *Drosophila* species show functional enhancer conservation and turnover during *cis*-regulatory evolution. Nat Genet 46: 685–692. 10.1038/ng.3009 24908250 PMC4250274

[GR276863DUC2] Bailey TL, Johnson J, Grant CE, Noble WS. 2015. The MEME Suite. Nucleic Acids Res 43: W39–W49. 10.1093/NAR/GKV416 25953851 PMC4489269

[GR276863DUC3] Banerji J, Rusconi S, Schaffner W. 1981. Expression of a β-globin gene is enhanced by remote SV40 DNA sequences. Cell 27: 299–308. 10.1016/0092-8674(81)90413-x 6277502

[GR276863DUC4] Bourque G, Leong B, Vega VB, Chen X, Lee YL, Srinivasan KG, Chew J-L, Ruan Y, Wei C-L, Ng HH, 2008. Evolution of the mammalian transcription factor binding repertoire via transposable elements. Genome Res 18: 1752–1762. 10.1101/gr.080663.108 18682548 PMC2577865

[GR276863DUC5] Britten RJ, Davidson EH. 1971. Repetitive and non-repetitive DNA sequences and a speculation on the origins of evolutionary novelty. Q Rev Biol 46: 111–138. 10.1086/406830 5160087

[GR276863DUC6] Cannavò E, Khoueiry P, Garfield DA, Geeleher P, Zichner T, Gustafson EH, Ciglar L, Korbel JO, Furlong EEM. 2016. Shadow enhancers are pervasive features of developmental regulatory networks. Curr Biol 26: 38–51. 10.1016/j.cub.2015.11.034 26687625 PMC4712172

[GR276863DUC7] Carter TA, Singh M, Dumbović G, Chobirko JD, Rinn JL, Feschotte C. 2022. Mosaic *cis*-regulatory evolution drives transcriptional partitioning of HERVH endogenous retrovirus in the human embryo. eLife 11: e76257. 10.7554/eLife.76257 35179489 PMC8912925

[GR276863DUC8] Chang CC, Chow CC, Tellier LCAM, Vattikuti S, Purcell SM, Lee JJ. 2015. Second-generation PLINK: rising to the challenge of larger and richer datasets. Gigascience 4: 7. 10.1186/S13742-015-0047-8/2707533 25722852 PMC4342193

[GR276863DUC9] Chaudhari HG, Cohen BA. 2018. Local sequence features that influence AP-1 *cis*-regulatory activity. Genome Res 28: 171–181. 10.1101/GR.226530.117 29305491 PMC5793781

[GR276863DUC10] Chuong EB, Elde NC, Feschotte C. 2016. Regulatory evolution of innate immunity through co-option of endogenous retroviruses. Science 351: 1083–1087. 10.1126/science.aad5497 26941318 PMC4887275

[GR276863DUC11] Doniger SW, Huh J, Fay JC. 2005. Identification of functional transcription factor binding sites using closely related *Saccharomyces* species. Genome Res 15: 701–709. 10.1101/GR.3578205 15837806 PMC1088298

[GR276863DUC12] Ellison C, Bachtrog D. 2013. Dosage compensation via transposable element mediated rewiring of a regulatory network. Science 342: 846–850. 10.1126/science.1239552 24233721 PMC4086361

[GR276863DUC13] The ENCODE Project Consortium, Moore JE, Purcaro MJ, Pratt HE, Epstein CB, Shoresh N, Adrian J, Kawli T, Davis CA, Dobin A, 2020. Expanded encyclopaedias of DNA elements in the human and mouse genomes. Nature 583: 699–710. 10.1038/s41586-020-2493-4 32728249 PMC7410828

[GR276863DUC14] Ernst J, Melnikov A, Zhang X, Wang L, Rogov P, Mikkelsen TS, Kellis M. 2016. Genome-scale high-resolution mapping of activating and repressive nucleotides in regulatory regions. Nat Biotechnol 34: 1180–1190. 10.1038/nbt.3678 27701403 PMC5125825

[GR276863DUC15] Fane M, Harris L, Smith AG, Piper M. 2017. Nuclear factor one transcription factors as epigenetic regulators in cancer. Int J Cancer 140: 2634–2641. 10.1002/ijc.30603 28076901

[GR276863DUC16] Feschotte C. 2008. Transposable elements and the evolution of regulatory networks. Nat Rev Genet 9: 397–405. 10.1038/nrg2337 18368054 PMC2596197

[GR276863DUC17] Grant CE, Bailey TL, Noble WS. 2011. FIMO: scanning for occurrences of a given motif. Bioinformatics 27: 1017–1018. 10.1093/BIOINFORMATICS/BTR064 21330290 PMC3065696

[GR276863DUC18] Hong JW, Hendrix DA, Levine MS. 2008. Shadow enhancers as a source of evolutionary novelty. Science 321: 1314. 10.1126/science.1160631 18772429 PMC4257485

[GR276863DUC19] Jacques PÉ, Jeyakani J, Bourque G. 2013. The majority of primate-specific regulatory sequences are derived from transposable elements. PLoS Genet 9: 1003504. 10.1371/journal.pgen.1003504 PMC3649963 23675311

[GR276863DUC20] Jang HS, Shah NM, Du AY, Dailey ZZ, Pehrsson EC, Godoy PM, Zhang D, Li D, Xing X, Kim S, 2019. Transposable elements drive widespread expression of oncogenes in human cancers. Nat Genet 51: 611–617. 10.1038/s41588-019-0373-3 30926969 PMC6443099

[GR276863DUC21] Katoh K, Misawa K, Kuma K, Miyata T. 2002. MAFFT: a novel method for rapid multiple sequence alignment based on fast Fourier transform. Nucleic Acids Res 30: 3059–3066. 10.1093/nar/gkf436 12136088 PMC135756

[GR276863DUC22] King MC, Wilson AC. 1975. Evolution at two levels in humans and chimpanzees. Science 188: 107–116. 10.1126/SCIENCE.1090005/ASSET/72CE3BB0-9EC2-4B40-9A60-8C0781D1AB65/ASSETS/SCIENCE.1090005.FP.PNG 1090005

[GR276863DUC23] Klein JC, Keith A, Agarwal V, Durham T, Shendure J. 2018. Functional characterization of enhancer evolution in the primate lineage. Genome Biol 19: 99. 10.1186/s13059-018-1473-6 30045748 PMC6060477

[GR276863DUC24] Klein JC, Agarwal V, Inoue F, Keith A, Martin B, Kircher M, Ahituv N, Shendure J. 2020. A systematic evaluation of the design and context dependencies of massively parallel reporter assays. Nat Methods 17: 1083–1091. 10.1038/s41592-020-0965-y 33046894 PMC7727316

[GR276863DUC25] Kuhn RM, Haussler D, James Kent W. 2013. The UCSC genome browser and associated tools. Brief Bioinform 14: 144–161. 10.1093/BIB/BBS038 22908213 PMC3603215

[GR276863DUC26] Kulakovskiy IV, Vorontsov IE, Yevshin IS, Sharipov RN, Fedorova AD, Rumynskiy EI, Medvedeva YA, Magana-Mora A, Bajic VB, Papatsenko DA, 2018. HOCOMOCO: towards a complete collection of transcription factor binding models for human and mouse via large-scale ChIP-Seq analysis. Nucleic Acids Res 46: D252–D259. 10.1093/NAR/GKX1106 29140464 PMC5753240

[GR276863DUC27] Kunarso G, Chia NY, Jeyakani J, Hwang C, Lu X, Chan YS, Ng HH, Bourque G. 2010. Transposable elements have rewired the core regulatory network of human embryonic stem cells. Nat Genet 42: 631–634. 10.1038/ng.600 20526341

[GR276863DUC28] Kwasnieski JC, Mogno I, Myers CA, Corbo JC, Cohen BA. 2012. Complex effects of nucleotide variants in a mammalian *cis*-regulatory element. Proc Natl Acad Sci 109: 19498–19503. 10.1073/pnas.1210678109 23129659 PMC3511131

[GR276863DUC29] Kwasnieski JC, Fiore C, Chaudhari HG, Cohen BA. 2014. High-throughput functional testing of ENCODE segmentation predictions. Genome Res 24: 1595–1602. 10.1101/gr.173518.114 25035418 PMC4199366

[GR276863DUC30] Löytynoja A. 2014. Phylogeny-aware alignment with PRANK. Methods Mol Biol 1079: 155–170. 10.1007/978-1-62703-646-7_10 24170401

[GR276863DUC31] Lynch VJ, Leclerc RD, May G, Wagner GP. 2011. Transposon-mediated rewiring of gene regulatory networks contributed to the evolution of pregnancy in mammals. Nat Genet 43: 1154–1159. 10.1038/ng.917 21946353

[GR276863DUC32] Maricque BB, Chaudhari HG, Cohen BA. 2019. A massively parallel reporter assay dissects the influence of chromatin structure on *cis*-regulatory activity. Nat Biotechnol 37: 90–95. 10.1038/nbt.4285 PMC7351048 30451991

[GR276863DUC33] McLeay RC, Bailey TL. 2010. Motif Enrichment Analysis: a unified framework and an evaluation on ChIP data. BMC Bioinformatics 11: 165. 10.1186/1471-2105-11-165 20356413 PMC2868005

[GR276863DUC34] Melnikov A, Murugan A, Zhang X, Tesileanu T, Wang L, Rogov P, Feizi S, Gnirke A, Callan CG, Kinney JB, 2012. Systematic dissection and optimization of inducible enhancers in human cells using a massively parallel reporter assay. Nat Biotechnol 30: 271–277. 10.1038/nbt.2137 22371084 PMC3297981

[GR276863DUC35] Moreau P, Hen R, Wasylyk B, Everett R, Gaub MP, Chambon P. 1981. The SV40 72 base repair repeat has a striking effect on gene expression both in SV40 and other chimeric recombinants. Nucleic Acids Res 9: 6047–6068. 10.1093/nar/9.22.6047 6273820 PMC327583

[GR276863DUC36] Nitta KR, Jolma A, Yin Y, Morgunova E, Kivioja T, Akhtar J, Hens K, Toivonen J, Deplancke B, Furlong EEM, 2015. Conservation of transcription factor binding specificities across 600 million years of bilateria evolution. eLife 4: e04837. 10.7554/eLife.04837 25779349 PMC4362205

[GR276863DUC37] Patwardhan RP, Lee C, Litvin O, Young DL, Pe'er D, Shendure J. 2009. High-resolution analysis of DNA regulatory elements by synthetic saturation mutagenesis. Nat Biotechnol 27: 1173–1175. 10.1038/nbt.1589 19915551 PMC2849652

[GR276863DUC38] Patwardhan RP, Hiatt JB, Witten DM, Kim MJ, Smith RP, May D, Lee C, Andrie JM, Lee S-I, Cooper GM, 2012. Massively parallel functional dissection of mammalian enhancers *in vivo*. Nat Biotechnol 30: 265–270. 10.1038/nbt.2136 22371081 PMC3402344

[GR276863DUC39] Pehrsson EC, Choudhary MNK, Sundaram V, Wang T. 2019. The epigenomic landscape of transposable elements across normal human development and anatomy. Nat Commun 10: 5640. 10.1038/s41467-019-13555-x 31822674 PMC6904449

[GR276863DUC40] Pollard KS, Hubisz MJ, Rosenbloom KR, Siepel A. 2010. Detection of nonneutral substitution rates on mammalian phylogenies. Genome Res 20: 110–121. 10.1101/GR.097857.109 19858363 PMC2798823

[GR276863DUC41] Purcell S, Neale B, Todd-Brown K, Thomas L, Ferreira MAR, Bender D, Maller J, Sklar P, de Bakker PIW, Daly MJ, 2007. PLINK: a tool set for whole-genome association and population-based linkage analyses. Am J Hum Genet 81: 559–575. 10.1086/519795 17701901 PMC1950838

[GR276863DUC42] Quinlan AR, Hall IM. 2010. BEDTools: a flexible suite of utilities for comparing genomic features. Bioinformatics 26: 841–842. 10.1093/BIOINFORMATICS/BTQ033 20110278 PMC2832824

[GR276863DUC43] Ramírez F, Ryan DP, Grüning B, Bhardwaj V, Kilpert F, Richter AS, Heyne S, Dündar F, Manke T. 2016. deepTools2: a next generation web server for deep-sequencing data analysis. Nucleic Acids Res 44: W160–W165. 10.1093/NAR/GKW257 27079975 PMC4987876

[GR276863DUC44] Schmidt D, Schwalie PC, Wilson MD, Ballester B, Gonçalves Â, Kutter C, Brown GD, Marshall A, Flicek P, Odom DT. 2012. Waves of retrotransposon expansion remodel genome organization and CTCF binding in multiple mammalian lineages. Cell 148: 335–348. 10.1016/j.cell.2011.11.058 22244452 PMC3368268

[GR276863DUC45] Siepel A, Bejerano G, Pedersen JS, Hinrichs AS, Hou M, Rosenbloom K, Clawson H, Spieth J, Hillier LDW, Richards S, 2005. Evolutionarily conserved elements in vertebrate, insect, worm, and yeast genomes. Genome Res 15: 1034–1050. 10.1101/GR.3715005 16024819 PMC1182216

[GR276863DUC46] Stampfel G, Kazmar T, Frank O, Wienerroither S, Reiter F, Stark A. 2015. Transcriptional regulators form diverse groups with context-dependent regulatory functions. Nature 528: 147–151. 10.1038/nature15545 26550828

[GR276863DUC47] Storer J, Hubley R, Rosen J, Wheeler TJ, Smit AF. 2021. The Dfam community resource of transposable element families, sequence models, and genome annotations. Mob DNA 12: 2. 10.1186/S13100-020-00230-Y 33436076 PMC7805219

[GR276863DUC48] Sundaram V, Cheng Y, Ma Z, Li D, Xing X, Edge P, Snyder MP, Wang T. 2014. Widespread contribution of transposable elements to the innovation of gene regulatory networks. Genome Res 24: 1963–1976. 10.1101/gr.168872.113 25319995 PMC4248313

[GR276863DUC49] Tewhey R, Kotliar D, Park DS, Liu B, Winnicki S, Reilly SK, Andersen KG, Mikkelsen TS, Lander ES, Schaffner SF, 2016. Direct identification of hundreds of expression-modulating variants using a multiplexed reporter assay. Cell 165: 1519–1529. 10.1016/J.CELL.2016.04.027 27259153 PMC4957403

[GR276863DUC50] Trizzino M, Park Y, Holsbach-Beltrame M, Aracena K, Mika K, Caliskan M, Perry GH, Lynch VJ, Brown CD. 2017. Transposable elements are the primary source of novelty in primate gene regulation. Genome Res 27: 1623–1633. 10.1101/gr.218149.116 28855262 PMC5630026

[GR276863DUC51] Ulirsch JC, Nandakumar SK, Wang L, Giani FC, Zhang X, Rogov P, Melnikov A, McDonel P, Do R, Mikkelsen TS, 2016. Systematic functional dissection of common genetic variation affecting red blood cell traits. Cell 165: 1530–1545. 10.1016/J.CELL.2016.04.048 27259154 PMC4893171

[GR276863DUC52] Vockley CM, Guo C, Majoros WH, Nodzenski M, Scholtens DM, Hayes MG, Lowe WL, Reddy TE. 2015. Massively parallel quantification of the regulatory effects of noncoding genetic variation in a human cohort. Genome Res 25: 1206–1214. 10.1101/GR.190090.115 26084464 PMC4510004

[GR276863DUC53] Wang T, Zeng J, Lowe CB, Sellers RG, Salama SR, Yang M, Burgess SM, Brachmann RK, Haussler D. 2007. Species-specific endogenous retroviruses shape the transcriptional network of the human tumor suppressor protein p53. Proc Natl Acad Sci 104: 18613–18618. 10.1073/pnas.0703637104 18003932 PMC2141825

[GR276863DUC54] Wray GA. 2007. The evolutionary significance of *cis*-regulatory mutations. Nat Rev Genet 8: 206–216. 10.1038/nrg2063 17304246

