# Supplementary material for: Functional characterization of enhancer activity during a long terminal repeat's evolution
Source: Genome Res. 2022 Oct;32(10):1840–51. doi: 10.1101/gr.276863.122 (PMC9712623; doi:10.1101/gr.276863.122)
Supplement: Supplemental Material [file supp_32_10_1840__DC1.html]

Functional characterization of enhancer activity during a long terminal repeat's evolution — Functional characterization of enhancer activity during a long terminal repeat's evolution — Supplemental Material 

# Functional characterization of enhancer activity during a long terminal repeat's evolution

## Supplemental Material

- Supplemental\_Data\_S1.zip
- Supplemental\_Data\_S2.zip
- Supplemental\_Table\_S1.xlsx
- Supplemental\_Table\_S2.xlsx
- Supplemental\_Table\_S3.xlsx
- Supplemental\_Table\_S4.xlsx
- Supplemental\_Table\_S5.xlsx
- Supplemental\_Table\_S6.xlsx
- Supplemental\_Code.zip
- Supplemental\_Methods.pdf
- Supplemental\_Figures.pdf
