# Supplementary material for: Functional characterization of enhancer activity during a long terminal repeat's evolution
Source: Genome Res. 2022 Oct;32(10):1840–51. doi: 10.1101/gr.276863.122 (PMC9712623; doi:10.1101/gr.276863.122)
Supplement: Supplemental Material [file supp_gr.276863.122_Supplemental_Methods.pdf]

Supplemental Methods:

LTR18A manual curation for ancestral reconstruction

We downloaded RepeatMasker 4.0.5 (Repeat Library 20140131) annotations for human (hg19), chimpanzee (panTro4), gorilla (gorGor3), gibbon (nomLeu3), rhesus macaque (rheMac3), and marmoset (calJac3) genomes (Smit et al.). For baboon (papAnu2) which is not available on www.repeatmasker.org, we ran RepeatMasker 4.1.0 using the RepBase RepeatMasker library 20170127. Since LTR18A consensus sequences are 98% similar between the two repeat libraries, we believe that most if not all LTR18A elements will be identified in papAnu2 in the same way as the other primate genomes. For the closest two subfamilies, LTR18B and LTR18C consensus sequences are ~75% and 67% similar to the LTR18A consensus respectively.

For manual curation, we examined the alignment of each annotated LTR18A element and removed the element if it satisfied any of our filtering criteria (Supplemental Table S3). First, we exclude LTR18A elements that have significant alignments to LTR18B or LTR18C. RepeatMasker outputs alignment scores for each repetitive element, some of which have multiple significant alignment scores for different subfamily consensus sequences. RepeatMasker then chooses the subfamily with the highest alignment score to annotate elements with the same ID. A consequence of this method is that fragmented elements can be annotated for the same subfamily even when the highest scoring alignment differs for each fragment. Since LTR18B and LTR18C consensus sequences are ~75% and 67% similar to LTR18A respectively, some LTR18A elements have significant alignments to LTR18B and/or LTR18C. Thus, we discard these elements with multiple possible alignments to avoid ambiguity from subfamily assignment. Second, we use paired LTR information to remove LTR18A elements that have discordant annotations. Due to the mechanism of ERV retrotransposition, we expect non-solo LTR18A elements to exist as same orientation pairs that are separated by the ERV internal region. Using this logic, we reasoned that paired LTRs that are assigned to different subfamilies have uncertain annotation.

Manual curation in LTR18A ancestral reconstruction

For manual curation of reconstructed ancestral sequences, we focused on insertions rather than deletions due to the possibility of insertions propagating up the tree. We determined insertion sites by examining the multiple sequence alignment of ortholog ancestors and finding gaps in the alignment created by insertions in only a few ortholog ancestors. Generally, we used parsimony when deciding to keep or remove an insertion. For example, if the insertion is present in only one primate lineage, then it is less likely for the insertion to have existed in the ortholog ancestor. Our reasoning is that an insertion in the ortholog ancestor and subsequent deletion in the other lineages requires at least two mutation events, whereas a single insertion in one primate lineage requires only one mutation event. Following reconstruction from ortholog ancestors, we again applied parsimony to manually adjust the LTR18A subfamily ancestor.

LTR18A MPRA library construction

Oligos described in Methods were ordered from Agilent and structured as follows: 5' priming sequence containing NheI site (CGGTATCTAAGAgctagcGT)/CRE/EcoRI site/Filler (if necessary)/BglII site/BamHI site/constant 'G'/barcode/constant 'A'/AgeI site/3' priming site (ATTAGCATGTCGTG) (Kwasnieski et al. 2014). Total length of oligos was 230bp.

The MPRA library was constructed as previously described with some adjustments. An AgeI site was introduced upstream of the SV40 polyA signal and the BamHI site downstream of the SV40 polyA signal was deleted using the QuikChange Lightning site-directed mutagenesis kit (Agilent). Synthesized oligos were amplified with 0.05pmol of template per 50μL PCR reaction for seven cycles using MPRA library amplification primers. A total of 32 reactions were performed. Following amplification and gel purification, oligos were cloned into a pGL backbone with the AgeI insert using NheI and AgeI sites. Multiple ligations were pooled, purified by PCR cleanup (Nucleospin), and transformed into 5-alpha electrocompetent *E. coli* (NEB). The hsp68 promoter driving dsRed reporter was cloned using EcoRI and BamHI sites. The MPRA library with the hsp68 promoter and dsRed reporter was purified and transformed into *E. coli* before plasmid extraction. The final library was concentrated by ethanol precipitation.

Cell culture and library transfection

Cell culture and library transfections were performed as previously described (Kwasnieski et al. 2014). K562 cells were grown in RPMI 1640 with L-glutamine (Gibco) + 10% Fetal Bovine Serum (FBS) + 1% penicillin/streptomycin. HepG2 cells were grown in Dulbecco's Modified Eagle Medium with high glucose, L-glutamine, and without sodium pyruvate + 10% FBS + 1% penicillin/streptomycin. For each of three replicates, 5 µg of library was transfected into 1.2 million cells using Neon electroporation (Life Technologies). For K562, electroporation parameters were three 10 millisecond pulses at 1450V. For HepG2, electroporation parameters were three 20 millisecond pulses at 1230V. As a transfection control, 0.5 µg of pmaxGFP (Lonza) was used.

Measurement of library expression

RNA extraction was performed 24 hours after transfection using PureLink RNA Mini Kit with on-column DNase treatment (Life Technologies) followed by DNase I treatment using TURBO DNA-free kit (Invitrogen). Samples were prepared for RNA-seq as previously described (Kwasnieski et al. 2014). First strand cDNA synthesis was performed using Superscript III Reverse Transcriptase (Life Technologies). Barcodes were amplified from cDNA from three transfections and three technical replicates of DNA from the plasmid library. Amplified barcodes were digested with KpnI and EcoRI and ligated to Illumina adapters. Ligation products were further amplified, after which replicates and plasmid library DNA input were pooled for sequencing. We obtained over 1000x average coverage for each transfection replicate and the DNA input.

MPRA evaluation

Enrichment scores of elements were highly reproducible across transfection replicates in HepG2 (average R^2=0.904) while moderately reproducible in K562 (average R^2=0.666) (Supplemental Figure S3). We confirmed that orientation does not have large effects on enrichment score in both HepG2 and K562

(Supplemental Figure S4). We also found that selected control sequences from Ernst et al. follow expected trends for both their original annotations as well as redefined annotations based on expression values from Ernst et al. MPRA results (Supplemental Figure S5).

Subfamily age estimate

The average divergence, weighted by copy length, was calculated for the LTR18A subfamily using the RepeatMasker output for hg19. The age was obtained by using the average divergence and the average mammalian genome mutation rate of 2.2×10^{-9} per base per year (Kumar and Subramanian 2002).

References

Kumar S, Subramanian S. 2002. Mutation rates in mammalian genomes. *Proc Natl Acad Sci* **99**: 803–808. doi:10.1073/PNAS.022629899.

Kwasnieski JC, Fiore C, Chaudhari HG, Cohen BA. 2014. High-throughput functional testing of ENCODE segmentation predictions. *Genome Res* **24**: 1595–602. doi:10.1101/gr.173518.114.

Smit A, Hubley R, Green P. RepeatMasker Open-4.0. *2013-2015 <http://www.repeatmasker.org>*.
