# Supplementary material for: Functional characterization of enhancer activity during a long terminal repeat's evolution
Source: Genome Res. 2022 Oct;32(10):1840–51. doi: 10.1101/gr.276863.122 (PMC9712623; doi:10.1101/gr.276863.122)
Supplement: Supplemental Material [file supp_gr.276863.122_Supplemental_Figures.pdf]

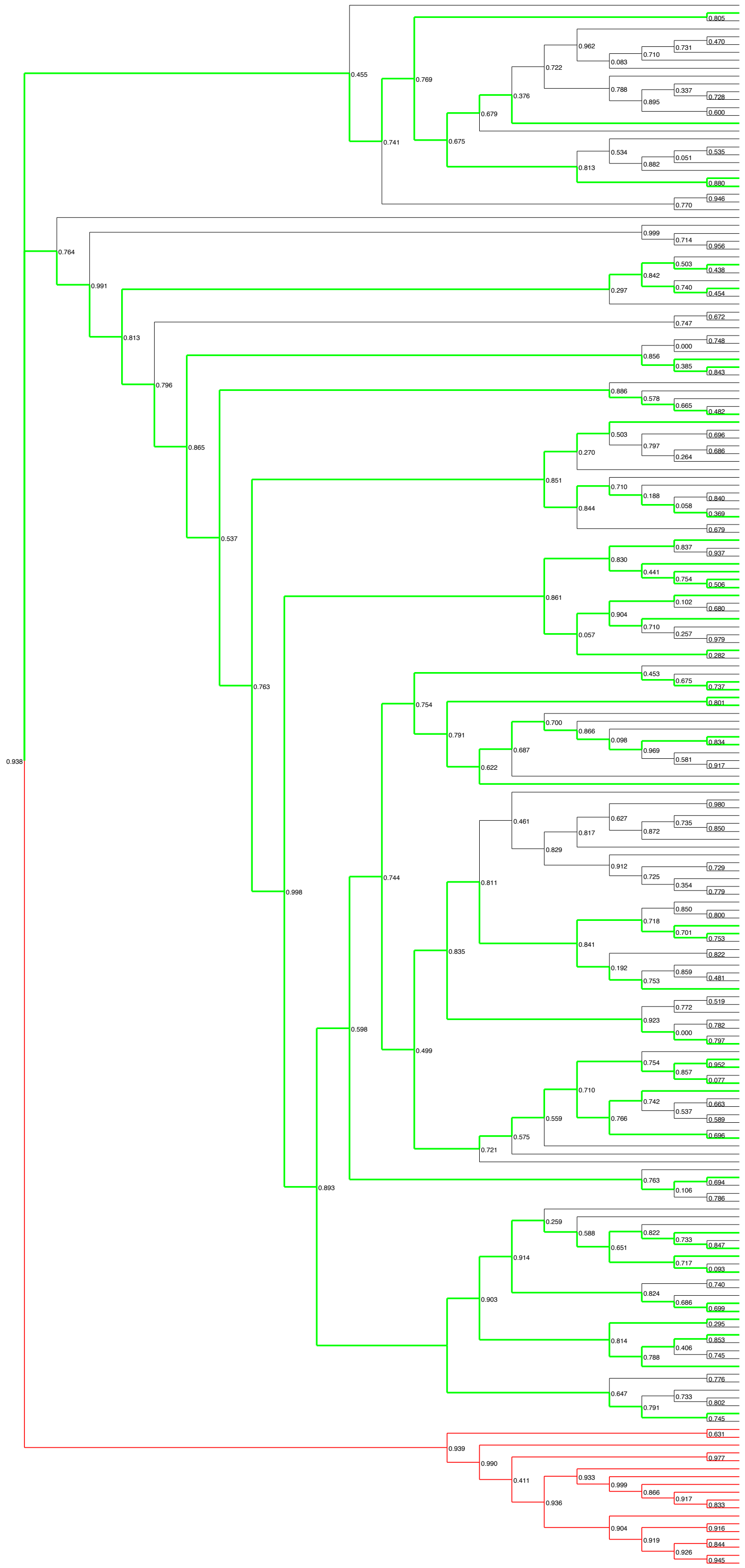

Supplemental Figure S1: Phylogenetic tree of manual curated hg19 LTR18A. Green lines indicate paths to hg19 LTR18A used in ancestral reconstruction. Red lines indicate paths to LTR18B elements used as an outgroup. Node labels are bootstrap values from FastTree.

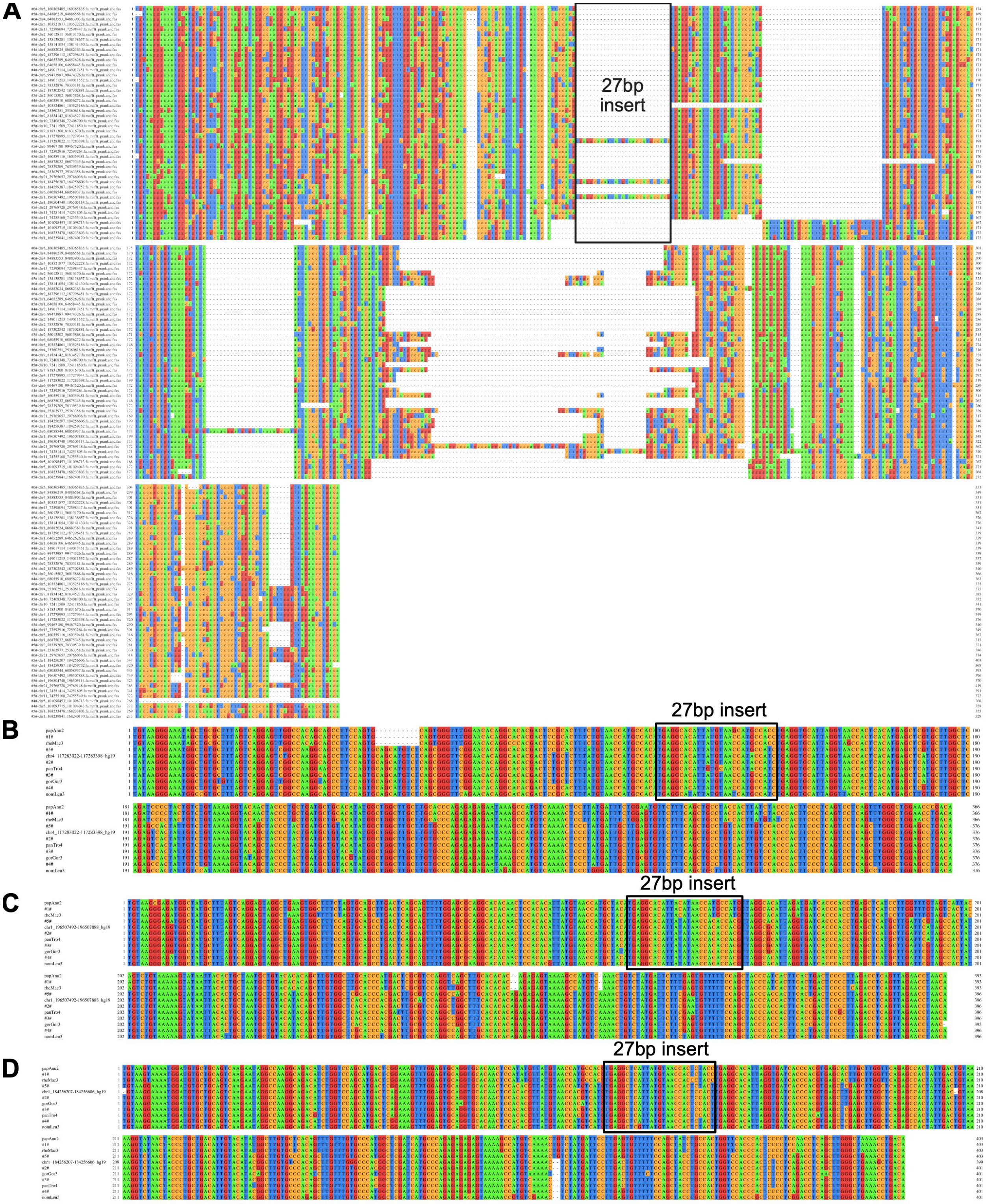

Supplemental Figure S2: MAFFT sequence alignment of LTR18A elements containing the 27bp insert. A) Alignment of all ortholog ancestors from the first round of ancestral reconstruction. The region of the 27bp insertion is boxed. B, C, and D) Alignments of the three sets of primate

orthologs and their reconstruction intermediates that contain the 27bp insertion (boxed for each alignment). Coordinates for the hg19 reference element are displayed.

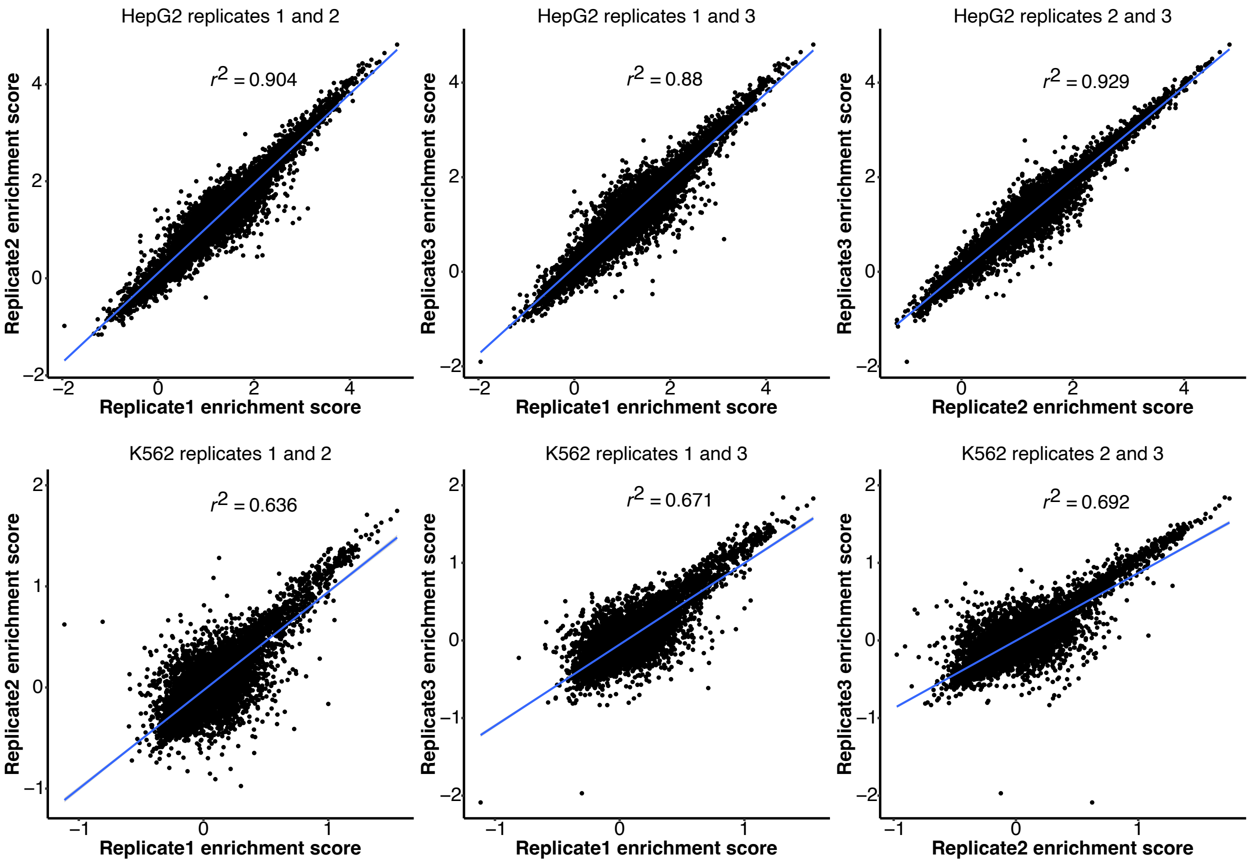

Supplemental Figure S3: Biological replicate enrichment score correlations for HepG2 and K562.

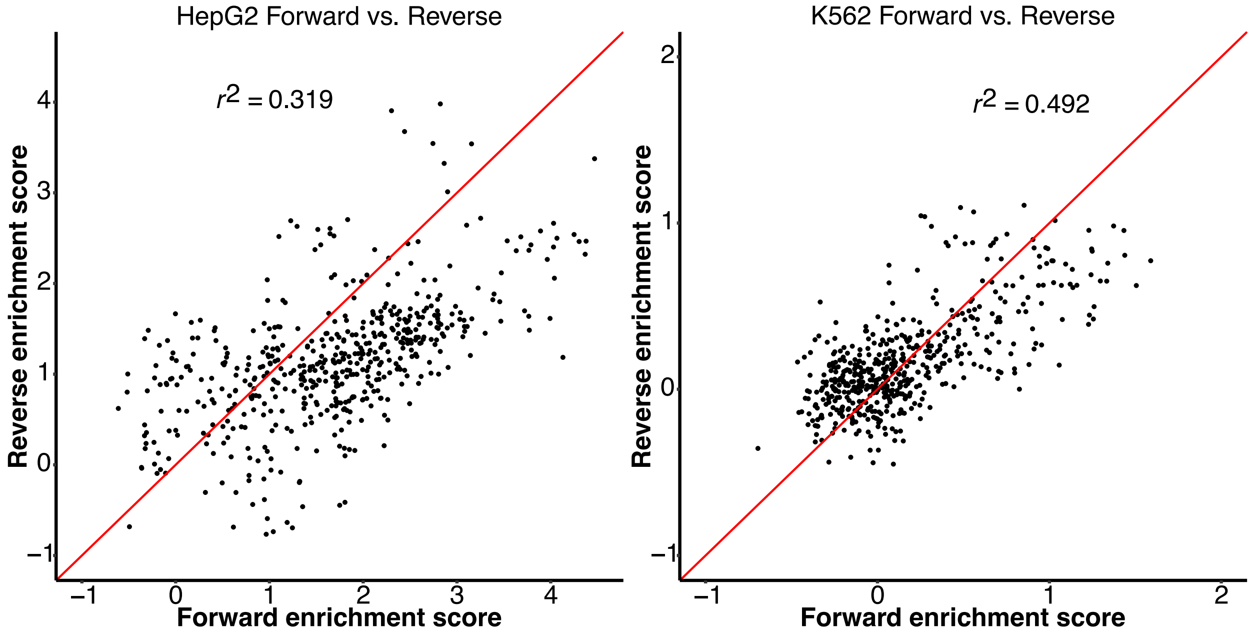

Supplemental Figure S4: Correlation of LTR18A fragments tested in forward and reverse orientations in HepG2 and K562.

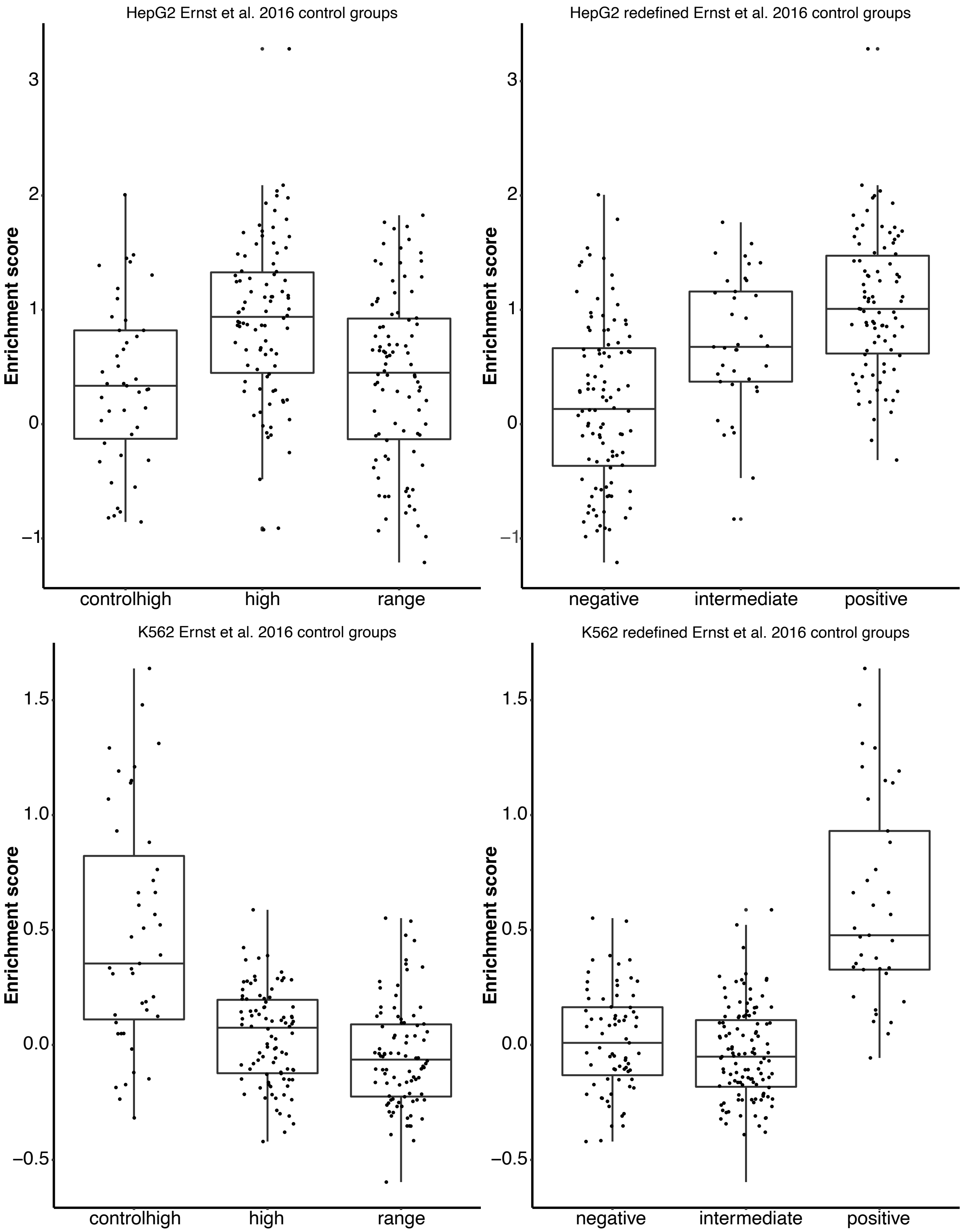

Supplemental Figure S5: Enrichment scores of controls from Ernst et al. 2016 in HepG2 and K562. Previously defined categories of sequences are as follows: “controlhigh” denotes candidate enhancer regions with strong H3K27ac dip scores in K562 but low activity states in

HepG2, “high” denotes candidate enhancer states in HepG2 with the strongest H3K27ac dip scores, “range” denotes regions with a range of dip scores. These same sequences were redefined to negative, intermediate, and positive groups based on their expression level in the Ernst et al. Pilot MPRA.

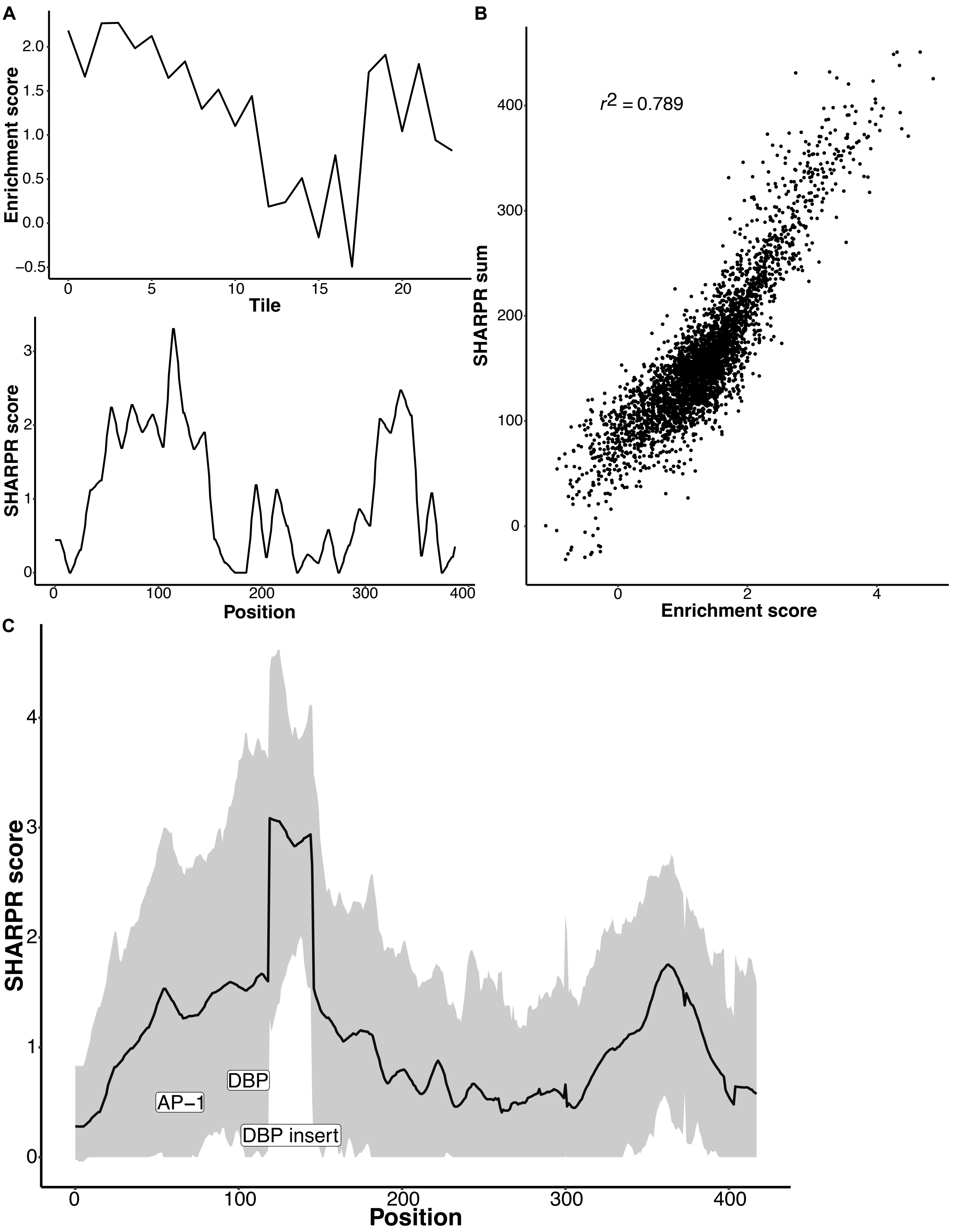

Supplemental Figure S6: SHARPR describes regulatory activity from tiling data. A) Transformation of tile enrichment scores (upper panel) to SHARPR nucleotide activity scores (lower panel) for the LTR18A subfamily reconstructed ancestor. B) Correlation of SHARPR

score sums with enrichment scores for every tile. C) SHARPR scores across tiled LTR18A elements. Elements were aligned to obtain universal positions. The mean SHARPR score for each position is plotted in the solid black line, with 90% of all scores falling within the grey shaded region. AP-1 and DBP motif positions are labeled, with the DBP motif in the 27bp insert differentiated from the consensus DBP motif.

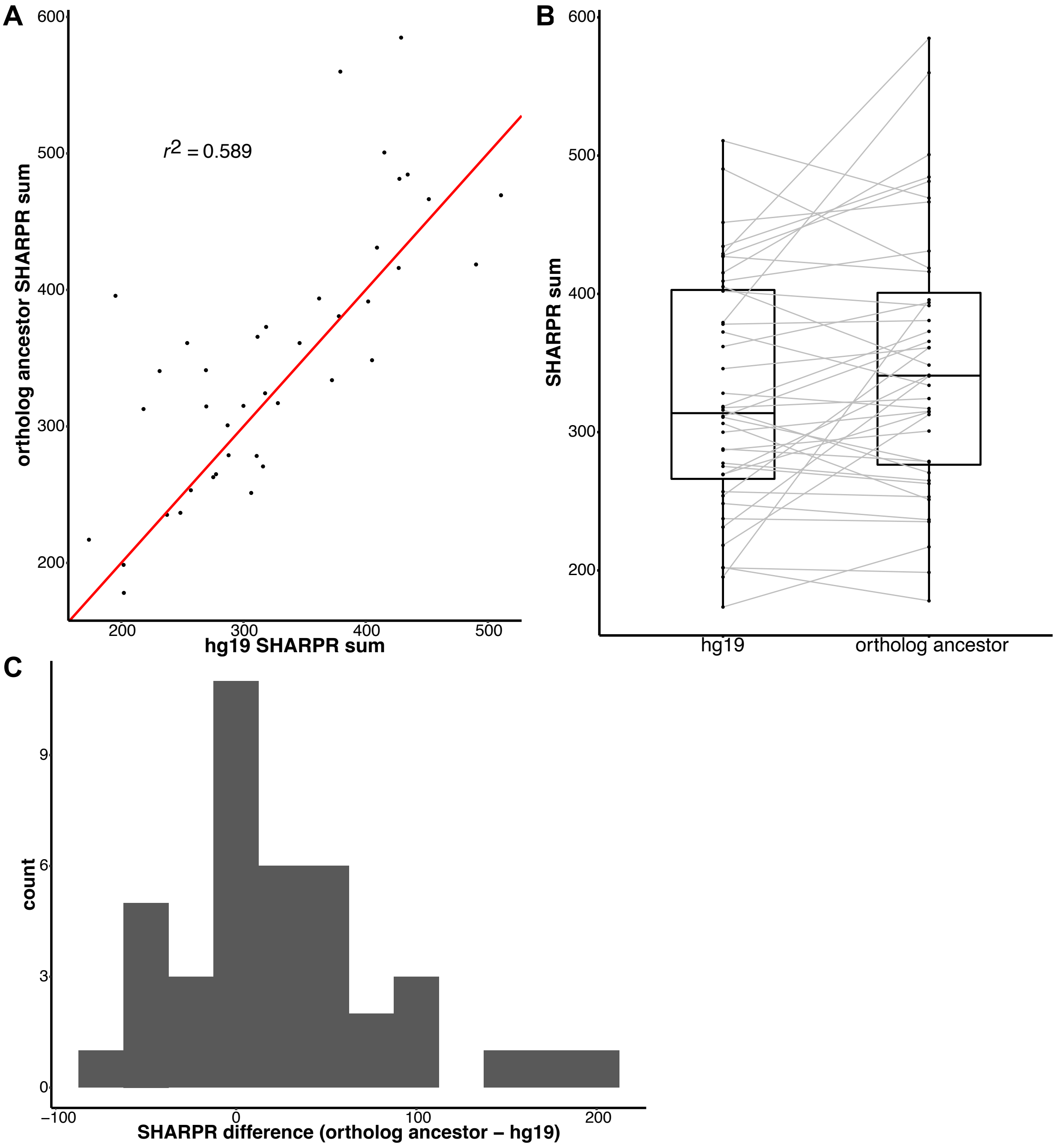

Supplemental Figure S7: Comparison of enhancer activities for present day hg19 LTR18A elements and their corresponding ortholog ancestors. A) Correlation of SHARPR sum for hg19 elements and their ortholog ancestors. The red line depicts a one-to-one relationship (y=x). B) Distribution of hg19 elements and ortholog ancestor SHARPR sums. Grey lines connect hg19 elements with their ortholog ancestor. C) Distribution of differences between ortholog ancestors and hg19 elements.

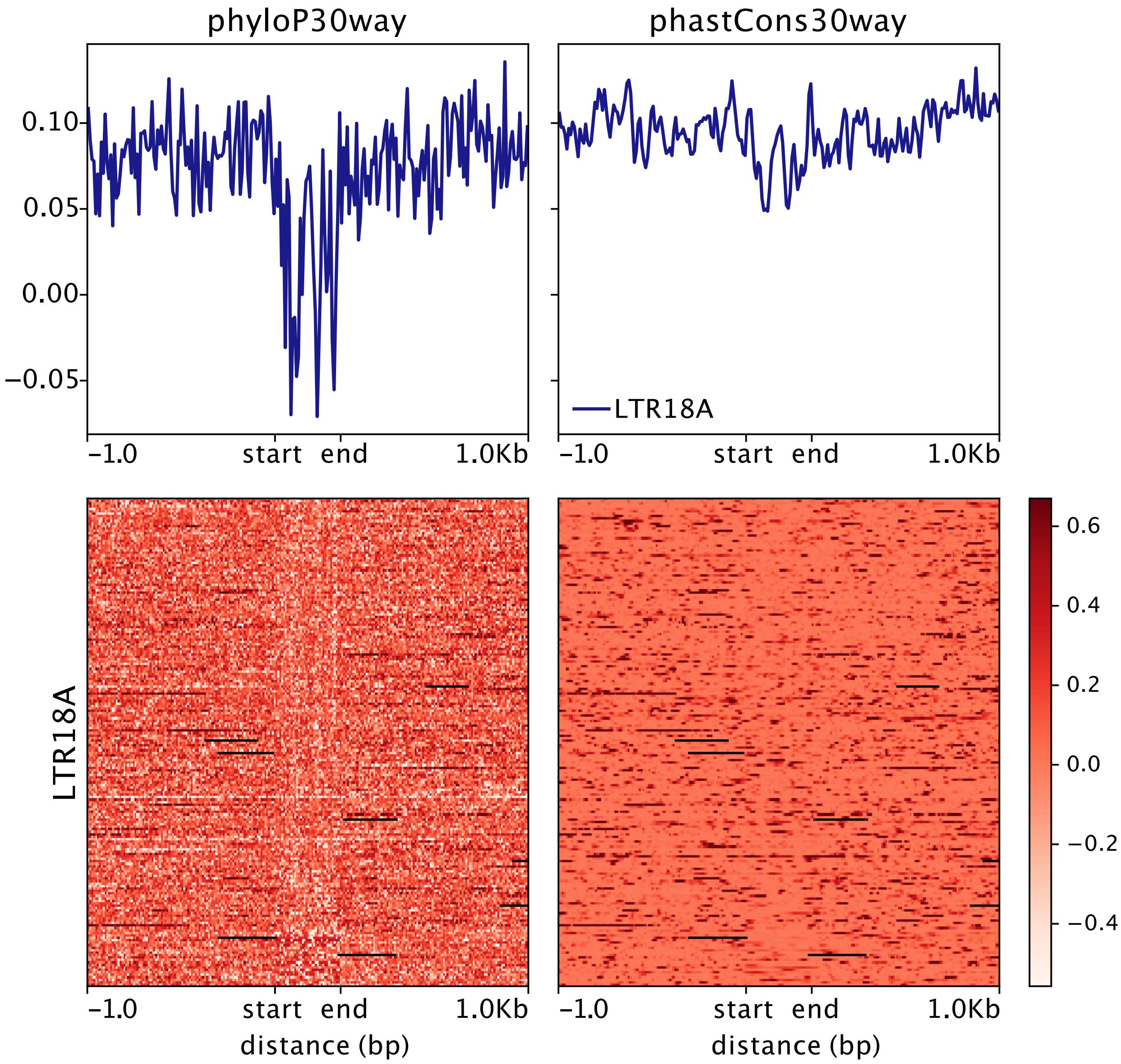

Supplemental Figure S8: Conservation scores for LTR18A and surrounding 1kb genomic regions. The phyloP and phastCons scores for 30 mammals (27 primates) are shown. Start and end refer to LTR18A start and end sites.

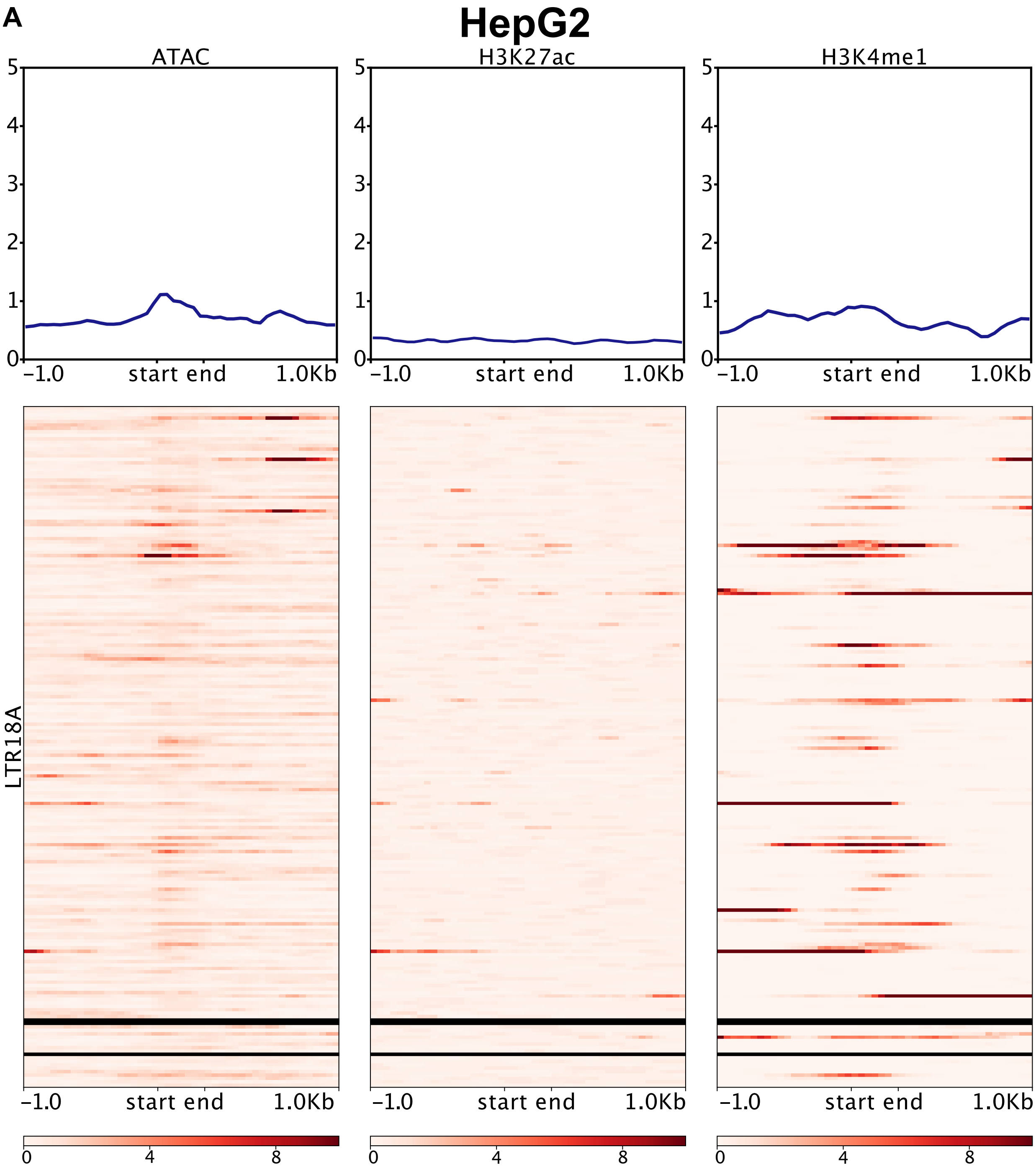

A
HepG2
ATAC
H3K27ac
H3K4me1
5
4
3
2
1
0
−1.0
start end
1.0Kb
LTR18A
0
4
8

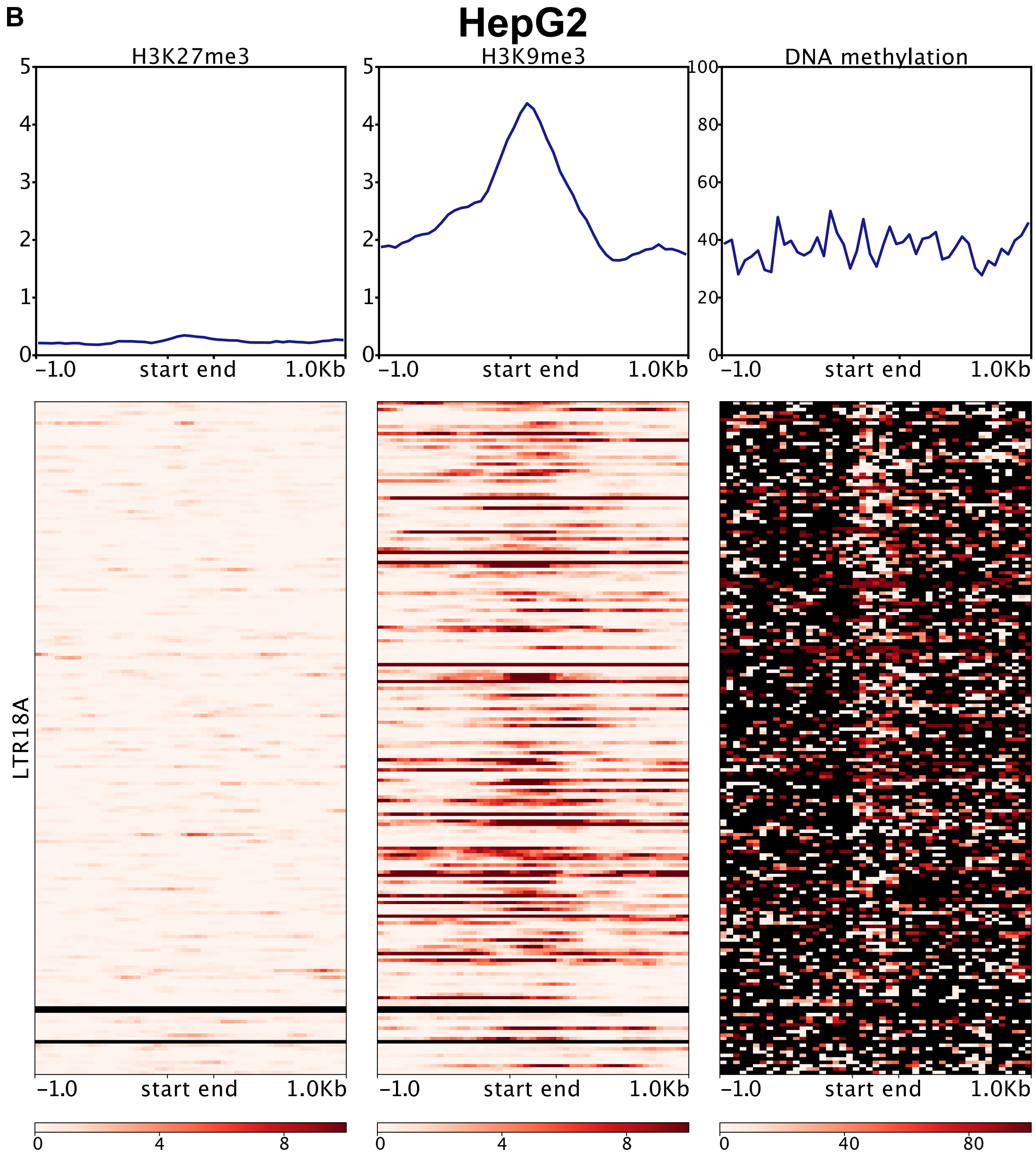
B
HepG2
H3K27me3
H3K9me3
DNA methylation
LTR18A
−1.0
start end
1.0Kb
0
4
8
40
80
100

C

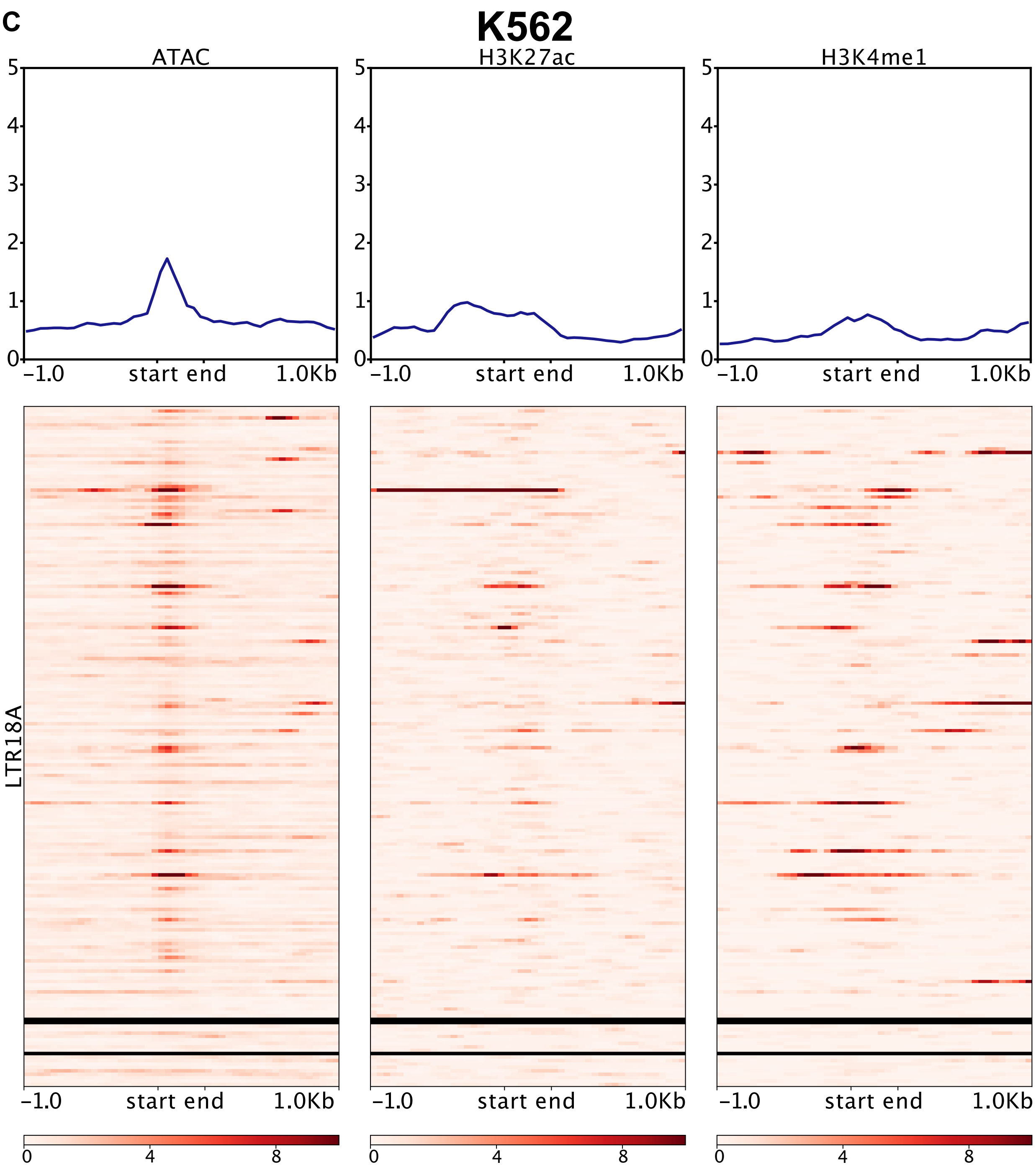

K562
ATAC
H3K27ac
H3K4me1
0
1
2
3
4
5
-1.0
start end
1.0Kb
LTR18A
0
4
8

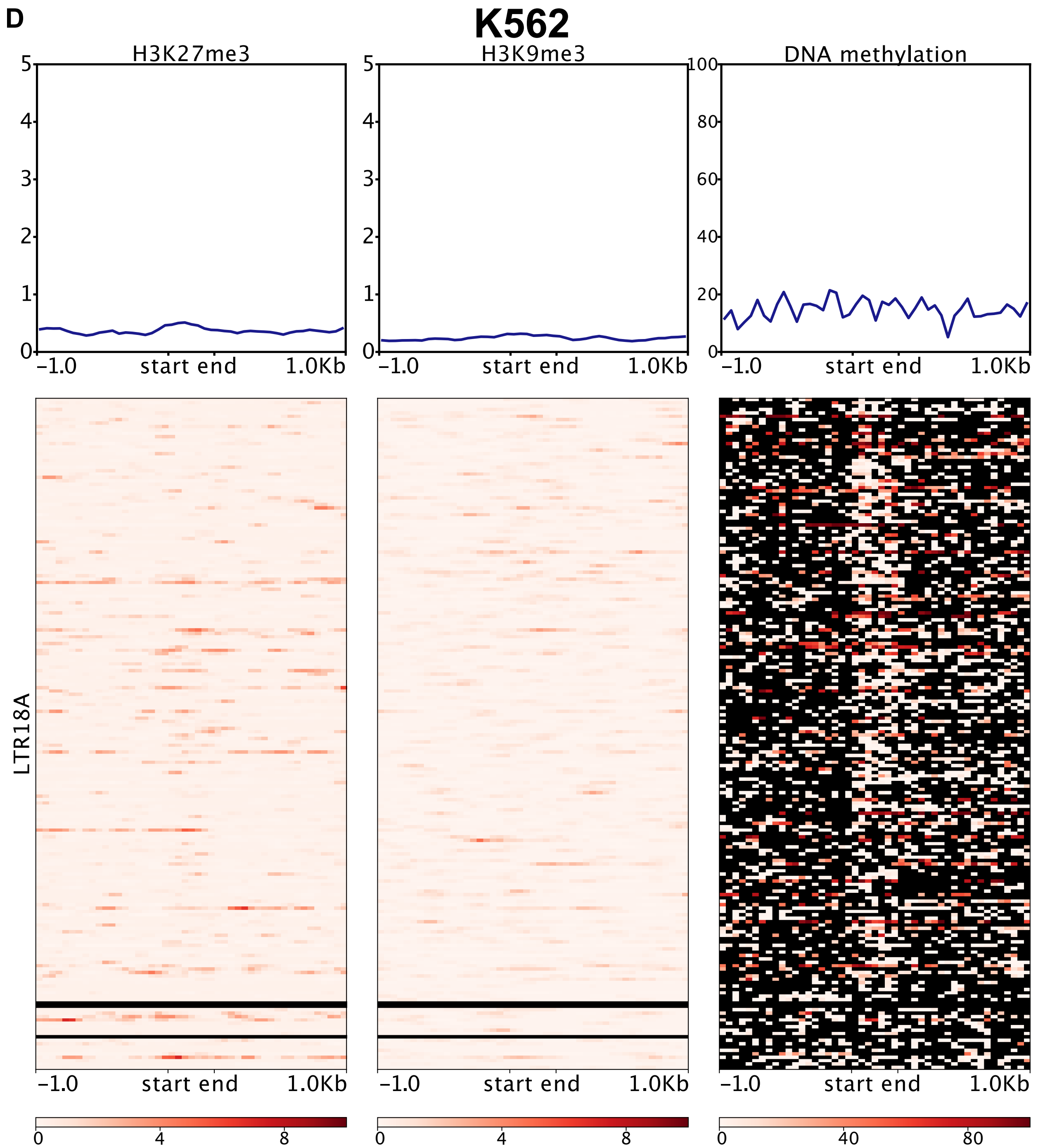

Supplemental Figure S9: Epigenetic marks at LTR18A in HepG2 (A and B) and K562 (C and D). Enhancer associated epigenetic marks (ATAC, H3K27ac, and H3K4me1) and repressive epigenetic marks (H3K27me3, H3K9me3, and DNA methylation) are shown. Three LTR18A elements have lengths shorter than the bin size (50bp) and are shown as black bars.

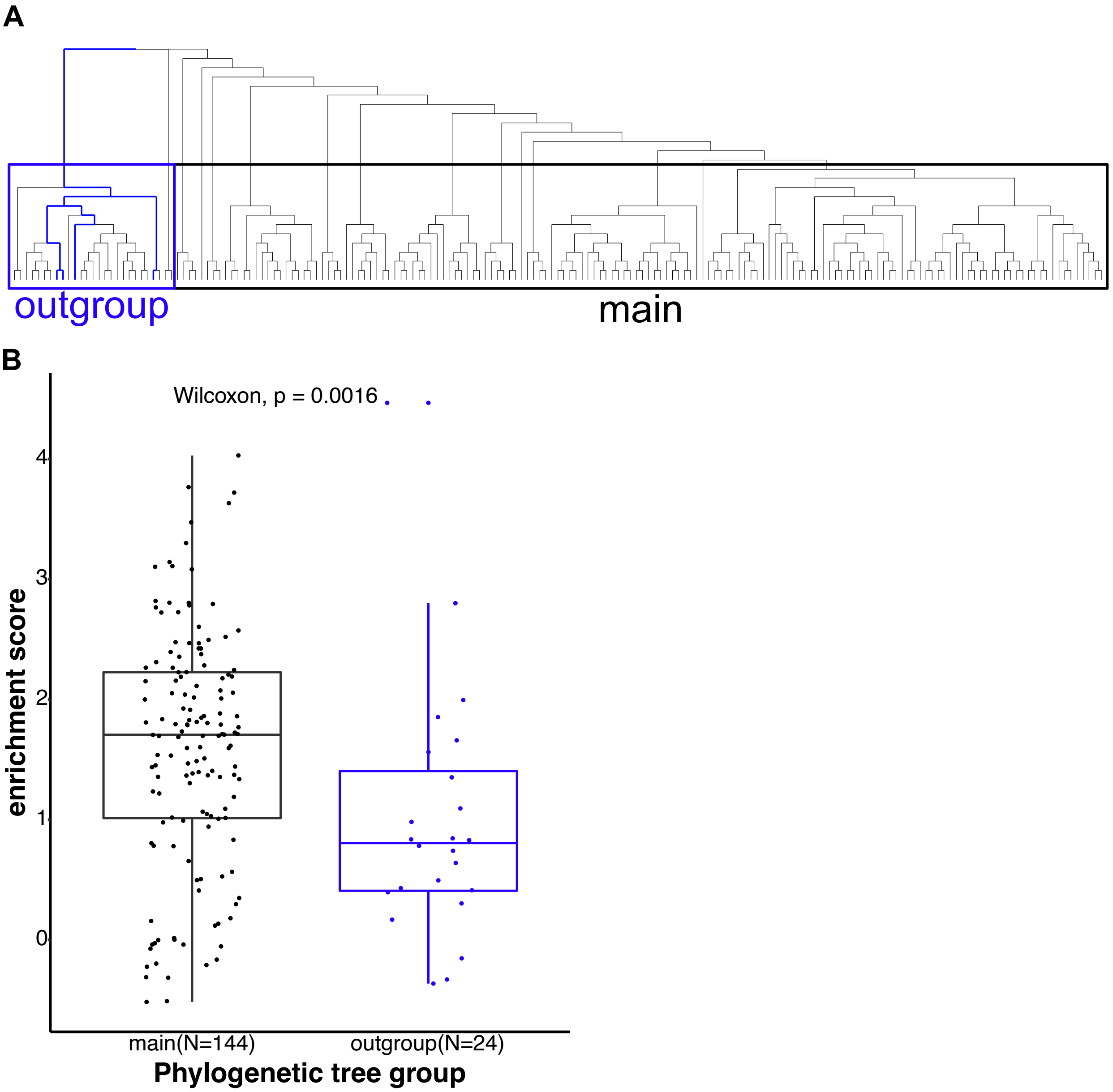

Supplemental Figure S10: Earliest diverging LTR18A lineage forms a functionally distinct outgroup. A) Phylogenetic tree of manual curated LTR18A splitting the earliest diverging lineage (outgroup, blue) from the main lineage (black). B) HepG2 enrichment scores of motif focused regions for main and outgroup hg19 LTR18A elements. *P* value derived from two-tailed Mann-Whitney *U* test.
